# Dairy farmers have minimal knowledge of bovine tuberculosis: A cross-sectional study in Bhutan

**DOI:** 10.1371/journal.pntd.0013817

**Published:** 2025-12-22

**Authors:** Juan-Pablo Villanueva-Cabezas, Sithar Dorjee, Justin McKinley, Rinzin Pem, Sangay Rinchen

**Affiliations:** 1 Department of Infectious Diseases, The University of Melbourne, at the Peter Doherty Institute for Infection and Immunity, Melbourne, Australia; 2 The Nossal Institute for Global Health, The University of Melbourne, Melbourne, Australia; 3 Khesar Gyalpo University of Medical Sciences, Thimphu, Bhutan; 4 Animal Health Division, Department of Livestock, Ministry of Agriculture and Livestock, Royal Government of Bhutan, Thimphu, Bhutan; 5 National Centre for Animal Health, Department of Livestock, Ministry of Agriculture and Livestock, Royal Government of Bhutan, Thimphu, Bhutan; 6 Gulbali Institute, Wagga Wagga, Faculty of Science and Health, Charles Sturt University, New South Wales, Australia; Christian Medical College Vellore, INDIA

## Abstract

Bovine tuberculosis (bTB) is a chronic disease of cattle and the leading cause of zoonotic tuberculosis. In Bhutan, there is no dedicated program for bTB despite the country being situated in the bTB enzootic region, where a large section of population relies on cattle and raw milk and, dairy products are widely consumed.To contribute to the design of future education, surveillance, mitigation, and control programs, we investigated the knowledge, attitudes, and practices relevant to bTB among dairy farmers. We surveyed 264 farmers in Thimphu, Paro, and Haa Dzongkhags. These farmers contribute to supplying the eight milk outlets in the capital, Thimphu. We found that only 11 farmers (4.2%; confidence interval 95%: 2.1% - 7.3%) were aware that bTB existed, and only five of them (1.9%; CI 95%: 0.6% – 4.3%) were aware that bTB is a zoonosis. Risk perception and practice followed a gradient of variation from Thimphu to Haa, but overall, we found a correlation between bTB risk perception for animals and humans. Farmers and traders, along with the consumption of meat and sick animals, were perceived as occupations and activities involving the highest risk of zoonotic infection. Contact with animals entering the herd and with neighbouring cattle were perceived as the highest risk for animal infection. Most farmers in Haa consume raw milk and dairy products they produced by themselves, whereas farmers in Paro and Thimphu prefer powdered milk. Using generalised low-rank models and k-means clustering, we found that dzongkhag of residence and attitudes toward zoonotic infection prevention explained most variability in the data. The severe knowledge deficits about bTB are particularly concerning given the zoonosis is present in Bhutan, is prevalent in neighbouring countries, and negatively affects cattle health and well-being, diminishing fertility, milk, and overall productivity, ultimately impacting farmers’ livelihoods and undermining Bhutan’s nutritional and economic reliance on this sector. Urgent short and mid-term activities should be prioritised to identify bTB high-risk areas, educate farmers, and mitigate bTB impacts.

## Author summary

This study investigated knowledge, attitudes, and practices regarding bovine tuberculosis (bTB)—a globally neglected zoonotic disease—among dairy farmers in Bhutan, a low- and middle-income country where raw milk and dairy products are food staples, religious restrictions prevent culling, and no public health or veterinary programs exist for the disease. The research surveyed 264 farmers across three dzongkhags (Thimphu, Paro, and Haa) who supply milk to the capital city. We found critically low awareness of bTB, with only 4.2% of farmers knowing its existence and 1.9% aware that this disease can be transmitted from animals to humans. Risk perception varied geographically. Farmers perceived new animals and cattle contact as primary risks for animal infection, while farming occupations and meat consumption were considered highest risk for human infection. Geographic location and zoonotic prevention practices explained most data variability. Given the high regional prevalence of bTB and recent confirmation of the disease among Bhutanese dairy herds, this knowledge gap necessitates urgent attention, and mitigation strategies to reduce the risk of healthy herds, individuals occupationally exposed to cattle, and those consuming dairy products must be developed and implemented.

## Introduction

Smallholder dairy farmers in low- and middle-income countries (LMICs) face significant challenges in managing bovine tuberculosis (bTB), a chronic infectious disease of cattle caused by *Mycobacterium bovis (M. bovis)* [1]. *M. bovis* spreads among cattle through daily farming practices, including close animal contact, shared feed and water sources, and exposure to contaminated bodily fluids [2]. Critically for farmers, *M. bovis* can also infect humans, primarily through consuming contaminated milk and dairy products and close contact with infected animals [3].

In Bhutan, small-scale livestock farming is an integral part of the rural economy. More than 60% of the population depends on agriculture and livestock farming for their livelihoods, and the small-scale dairy operation is a longstanding traditional practice [4–6]. Traditionally, cattle rearing relied on extensive free grazing in forests during the day with animals brought into sheds at night [7]; more recently, there has been a shift towards more intensive systems that involve indoor stall feeding, most common in lower altitudes, and the introduction of improved cattle varieties like Brown Swiss and Jersey [8]. Dairy operations have experienced significant market-oriented growth since the late 1990s in response to local demand for dairy products, predominantly manufactured by smallholder farmers, often using unpasteurised milk [9]. Despite the considerable expansion of dairy production in Bhutan and most of the region [10], smallholder dairy farmers frequently struggle with limited access to biosecurity, production technologies and formal markets, compromising their ability to implement effective disease control measures [1,3]. Many small-scale dairy farmers still milk their animals manually, with negative effects on animal welfare and milk safety [11].

The recent confirmation of *M. bovis* seropositivity among dairy herds in Bhutan [12] reflects a broader regional challenge: *M. bovis* is known to be endemic across Southeast Asia, but its prevalence in cattle populations and milk supplies remains poorly documented as most countries do not have surveillance programs [13]. This surveillance gap is of concern as the region carries the highest global burden of human tuberculosis [14], and the specific contribution of *M. bovis* to this burden remains largely unquantified. Where surveillance data exists, prevalence varies dramatically across the region; Bhutan’s recent study in six eastern districts found a true seroprevalence of 0.91% [12], while the neighbouring state of Assam, India reported rates of 28% [15], more than double the global prevalence rate of 13.12%[16].These surveillance challenges are compounded by low disease awareness among farming communities; a systematic review of Asian farmers [17] revealed a critical disconnect between risk behaviours and disease knowledge: while many frequently consume unpasteurised products (range between 23% – 70% of farmers), their awareness of bTB remains remarkably low (3% – 33%), with limited understanding of zoonotic transmission through respiratory droplets (0% – 17%) or milk consumption (14% – 37%).

The risk of bTB spread in Bhutan is influenced by unique cultural norms and agricultural practices. Buddhist traditions of *tsethar* (saving lives of animals destined for slaughter) and *ahiṃsā* (non-violence) mean livestock keepers rarely kill their cattle for meat, allowing them to die naturally and resulting in larger herd sizes with older cattle that are more susceptible to bTB infections [18,19]. Traditional grazing practices, particularly free-range systems and transhumance (seasonal migration), provide opportunities for cattle mixing from different households and locations, potentially facilitating disease spread across herds and geographic regions [20,21]. Despite the identified risks and regional burden, Bhutan currently lacks a comprehensive bTB surveillance program for animals, food, or people. While veterinary services are delivered through the Department of Livestock’s and its network of animal health facilities instituted at National, regional, district and sub-district level, significant operational challenges—including inconsistent diagnostic quality and challenging sample transport in remote areas—impede effective bTB surveillance implementation [22]. To partially overcome these limitations and inform appropriate control strategies, cross-sectional knowledge, attitudes, and practices (KAP) study can provide valuable insights to support the development of culturally appropriate and evidence-driven advocacy, communication, and community engagement strategies. These insights are crucial for achieving sustainable social and behavioural change [23] that mitigate the spread of bTB among herds and reduce zoonotic infection risk. Thus, this study aimed to investigate Bhutanese dairy farmers’ knowledge, attitudes, and practices towards bTB.

## Methods

### Ethics and reporting

This study received ethical approval from the Human Ethics Committee of the University of Melbourne (Ethics ID: 023-25948-39668-4) and The Livestock Technical Advisory Committee of the Department of Livestock, Ministry of Agriculture and Livestock, Bhutan (Meeting 9 March 2023). The study is reported in accordance with the Strengthening the Reporting of Observational Studies in Epidemiology – veterinary extension guidelines (STROBE-VET) [24].

### Study area and population

**Fig 1:**
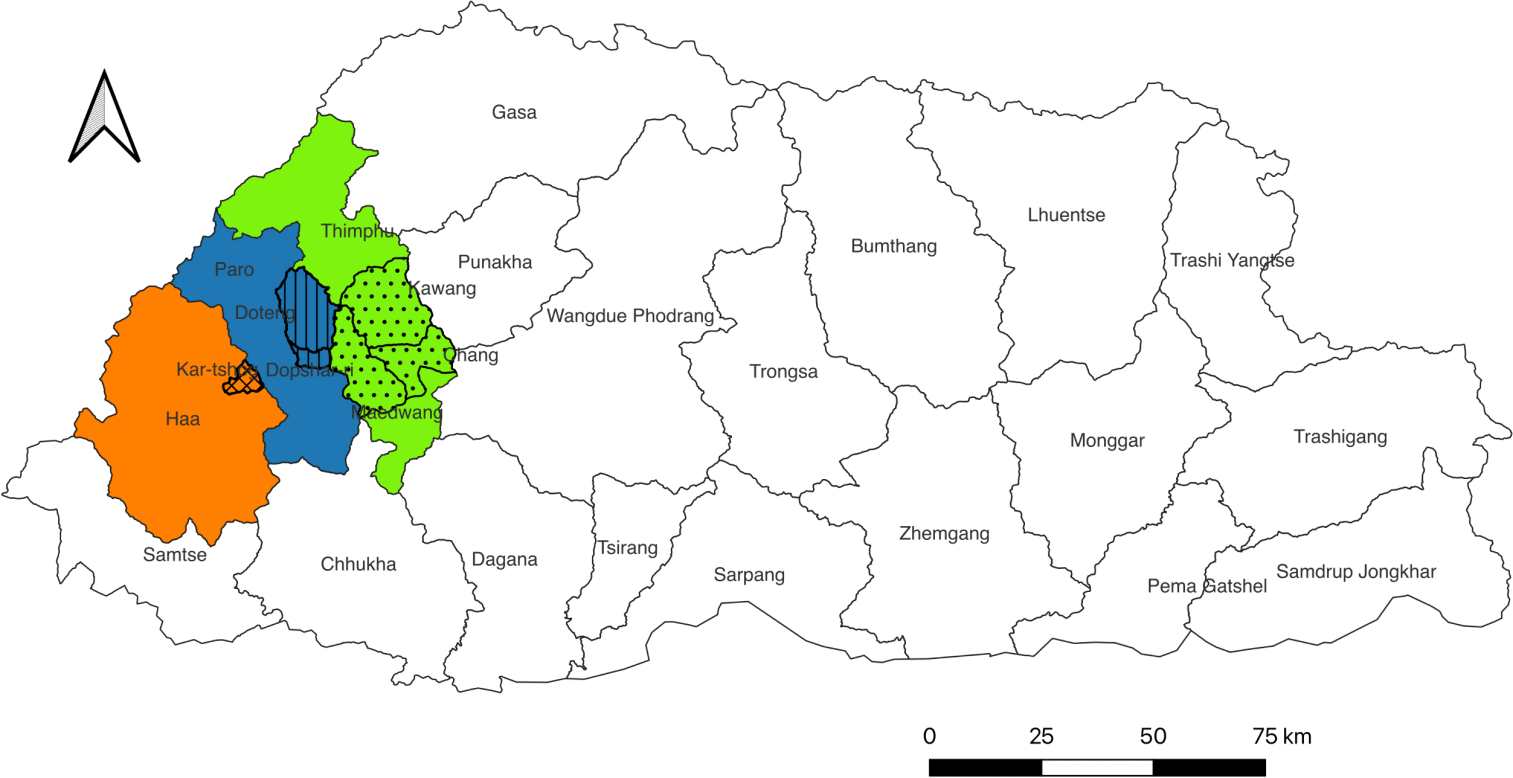
Showing the study areas Kawang, Chang and Mewang geogs under Thimphu Dzongkhag; Doteng and Dopshari geogs under Paro; and Katsho geog under Haa. The map was produced using Quantum Geographical Information System (QGIS) software version 3.24 [26]. The shapefiles of the administrative boundaries of Bhutan and its districts and subdistricts were obtained from DIVA-GIS (https://diva-gis.org/data.html#google_vignette).

The Kingdom of Bhutan (capital: Thimphu) is a landlocked country in the eastern Himalayas, administratively divided into 20 dzongkhags (districts) and 205 geogs (subdistricts or village groups). The most recent national livestock census enumerated 54,149 livestock holders and a cattle population estimated at 295,444 heads, including improved milch populations of Jersey, Brown Swiss, and Holstein Friesian, increasingly preferred by smallholders [25]. The same census recorded the production of 54,654 metric tons (MT) of milk, 1,930 MT of butter, 3,154 MT of cheese, and 198 MT of chugo (traditional Yak cheese). Our study was conducted in six geogs across three dzongkhags of Bhutan that contribute to Thimphu’s milk and dairy product supply: Katsho geog in Haa (293 km from Thimphu), Dopshari and Doteng geogs in Paro (51 km from Thimphu), and Chang, Kawang and Mewang geogs in Thimphu itself. The target population was the dairy farmers supplying Thimphu’s milk value chain. The source population for our sample consisted of farmers with membership in dairy cooperatives that supplied milk and dairy products to the eight milk outlets in Thimphu, as per the records of the Department of Livestock. A map of the study area is provided in Fig 1.

### Study design and sample size

We designed a cross-sectional survey to investigate farmers’ knowledge, attitudes, and practices (KAP) on bTB. The questions were developed based on data from a systematic review of KAPs towards bovine tuberculosis in LMICs [17] and expert input from Bhutanese livestock veterinarians and public health professionals to ensure locally relevant aspects were captured. The survey was tested and validated by researchers from Bhutan’s National Centre for Animal Health, staff from the Department of Livestock (veterinarians, para-veterinarians), researchers from Khesar Gyalpo University of Medical Sciences and the University of Melbourne. The final version of the survey was translated into Dzongkha, the official language of Bhutan (Appendix A in S1 Text).

Considering that farmers’ bTB awareness ranges from 3% to 33% in Asia, and this group is usually aware of human tuberculosis [17], we estimated that 20% of the farmers would answer positively to the question *“Before this survey, did you know about bTB?”.* Therefore, a sample of at least 246 farmers was needed to estimate the overall farmers’ bTB awareness within 5% of the true population value [27].

### Data collection

The KAP survey was integrated into REDCap software [28] to facilitate digital, encrypted data collection. Five public health students from Khesar Gyalpo University of Medical Sciences Bhutan and one senior para-veterinarian were recruited and trained by JP Villanueva-Cabezas to perform data collection using the mobile phone REDCap application (V5.24.7). The public health students collected data in Paro (two surveyors), Thimphu (three surveyors), while the para-veterinarian collected data in Haa. S.Rinchen, R. Pem, and the local para-veterinarians contacted the milk cooperatives to inform them about the study and its objectives. When cooperatives agreed to participate, a day and time were arranged for the surveys to be carried out in person at the local livestock extension centre.

The surveys were conducted between July 10 and 14, 2023, under the supervision of S. Rinchen, S. Dorjee, and JP Villanueva-Cabezas. Farmers were received with refreshments and invited to participate in the study. After obtaining verbal informed consent, surveys were conducted in person, individually for a median duration of 12 minutes. The collected data was transferred in real-time to an encrypted server hosted by the University of Melbourne.

### Statistical analysis

#### Descriptive analysis.

We used R software version 4.3.0 [29] to assess, clean, and perform descriptive analyses on the data. The analysis was performed on the entire sample and stratified by dzongkhag to provide a more nuanced understanding of the data. Key results were presented in tables and frequency plots. All results are tabulated and presented in Appendix B in S1 Text.

#### Dimensional reduction and clustering.

We investigated the structure of the farmers’ data using a two-step approach. First, we reduced the dimensionality of the data using Generalised Low-Rank Models (GLRM), a generalisation of standard matrix factorisation that can incorporate various data forms and capture non-linear relationships [30]. The models were built using H2O [31]—an in-memory environment for building machine learning models in R. To determine the number of GLRM-derived archetypes (“principal components”) needed to characterise the variation of the data, we used the scree plot of the eigenvalues for each archetype [30]. Second, we used K-means clustering [32] on the decomposed matrix generated by GLRM and determined, using the elbow test [33], the ideal number of clusters needed to group and characterise the farmers in our dataset. Clusters were characterised. Details are presented in Appendix C in S1 Text.

#### Correlations of risk perception.

Respondents were asked 14 questions about bTB risk perception: eight about human bTB risk and six about animal bTB risk. We used these questions to create indices to investigate correlations between the respondents’ perceived bTB risks for humans and animals. The indices were created as follows:


$$I_{human}=\frac{\sum_{h=\text{1}}^{\text{8}}X_{hi}}{\text{8}},$$ (1)

where X=(1 low risk,  2 medium risk,  3 high risk), h is a series of eight human bTB risk variables: 1.) consuming milk, 2.) consuming dairy products, 3.) eating meat, 4.) consuming an animal with signs of bTB, 5.) contact with animals, 6.) contact with people, 7.) allowing cattle into dwelling, and 8.) sharing a water source with your cattle for each individual i.

We repeated this process for bTB risk in animals such that


$$I_{animal}=\frac{\sum_{a=\text{1}}^{\text{6}}X_{ai}}{\text{6}},$$ (2)

where a is a series of six animal bTB risk variables: 1.) bringing new cattle into the herd, 2.) contact between neighbouring cattle and own cattle, 3.) contact between own cattle and veterinarians, 4.) contact between own cattle and wildlife, 5.) contact between own cattle and other livestock species, and 6.) contact between own cattle and dogs for each individual i.

The total range allowed in the two indices is from a minimum of one to a maximum of three. Thus, one would be the index score for an individual reporting *low risk* for all responses and three for an individual reporting *high risk* for all responses. The higher the score, the greater the risk perception captured by the human index ($I_{humani})$ and the animal index ($I_{animal}$).

## Results

A total of 273 farmers agreed to participate in the survey, of which nine were excluded: three underage participants who wanted to complete the survey on behalf of their parents, two farmers who sold all their animals in the months preceding the study, and four participants who completed the survey but had missing responses to the question “D*id you know about bovine tuberculosis before this survey?”* A total of 264 farmer observations is reported.

Most farmers in our study resided in Haa (50.4%, n = 133), followed by Thimphu (32.2%, n = 85) and Paro (17.4%, n = 46). Overall, the median age of farmer was 50 years (Interquartile range [IQR] 40–60), with dzongkhag-specific medians ranging from 45 years in Thimphu to 53 years in Haa. Most respondents were women and household heads. The educational attainment of participants was low, with about half of the farmers reporting no formal education (55.3%, 95% CI: 49.1%–61.4%). The median household size was four members (IQR:3–5), and farmers owned a median of five cattle (IQR: 3–7) (Table 1).

**Table 1:** Characteristics of study participants.

Characteristic	Overall	Haa, n = 133	Paro, n = 46	Thimphu, n = 85
**Sex** *n (%)*				
Male	93 (35.2%; 95% CI: 29.4%–41.4%)	34 (25.6%)	25 (54.3%)	34 (40.0%)
Female	171 (64.8%; 95% CI: 58.6%–70.6%)	99 (74.4%)	21 (45.7%)	51 (60.0%)
**Age** *median (IQR)*	50.0 (40, 60)	53.0 (45, 61)	51.0 (37, 61)	45.0 (35, 59)
**Education** *n (%)*				
Primary	51 (19.3%; 95% CI: 14.7%–24.6%)	23 (17.3%)	8 (17.4%)	20 (23.5%)
Secondary	30 (11.4%; 95% CI: 7.8%–16.0%)	6 (4.5%)	10 (21.7%)	14 (16.5%)
Tertiary	25 (9.5%; 95% CI: 6.2%–13.7%)	17 (12.8%)	2 (4.3%)	6 (7.1%)
Monastic/ Non formal	12 (4.5%; 95% CI: 2.4%–7.8%)	4 (3.0%)	3 (6.5%)	5 (5.9%)
None	146 (55.3%; 95% CI: 49.1%–61.4%)	83 (62.4%)	23 (50.0%)	40 (47.1%)
**Household head** *n = 263 (%)*	177 (67.3%; 95% CI: 61.2%–73.0%)	102 (76.7%)	26 (57.8%)	49 (57.6%)
**Household size** *median (IQR)*	4.0 (3, 5)	5 (3, 5)	4.0 (3, 5)	4.0 (4, 5)
**Cattle owned** *median (IQR)*	5.0 (3, 7)	5 (3, 6)	4.0 (3, 6)	5.0 (4, 8)

### Knowledge about bTB

Pre-existing knowledge of bovine tuberculosis was almost non-existent, with 95.8% of participants (95% CI: 92.8%–97.9%) reporting no prior awareness of the disease. Out of the 11 who were aware of bTB, only five knew that bTB is a zoonosis (1.9%; 95% CI: 0.6%–4.3%) (Table 2). Most farmers did not know the sources of bTB infection in humans or cattle, or the signs shown by cattle infected with bTB. Farmers guessed that direct contact with infected cattle and consumption of unpasteurised products were the primary sources of zoonotic infection. Similarly, farmers guessed that contact with infected cattle was the main source of bTB infection for susceptible cattle, and that weight loss and respiratory signs were indicators of bTB in cattle. A significant number of farmers guessed that cattle infected with bTB would appear unhealthy, have a shortened lifespan, and would not recover without treatment (Appendix B in S1 Text).

**Table 2:** Knowledge of bovine tuberculosis and human tuberculosis among Bhutanese farmers.

Questions	Response	Overall (frequency, 95% CI)**	Haa	Paro	Thimphu
**Pre-existent bTB knowledge**					
Know bTB and zoonotic potential	--	5 (1.9%; CI: 0.6%–4.3%)	1 (0.8%)	2 (4.3%)	2 (2.4%)
Know bTB only	--	6 (2.3%; CI: 0.8%–4.9%)	1 (0.8%)	2 (4.3%)	3 (3.5%)
Don’t know bTB	--	253 (95.8%; CI: 92.8%–97.9%)	131(98.5%)	42 (91.3%)	80 (94.1%)
**PEOPLE infected with any type of tuberculosis…**					
may look healthy	Yes	30 (11.4%; CI: 7.8%–16.0%)	24 (18.0%)	5 (10.9%)	1 (1.2%)
	No	212 (80.6%; CI: 75.3%–85.2%)	99 (74.4%)	30 (65.2%)	83 (98.8%)
	don’t know	21 (8.0%; CI: 5.0%–12.1%)	10 (7.5%)	11 (23.9%)	0 (0.0%)
	Missing	1	0	0	1
can be cured	Yes	176 (66.9%; CI: 60.9%–72.6%)	89 (67.4%)	33 (71.7%)	54 (63.5%)
	No	58 (22.1%; CI: 17.1%–27.7%)	28 (21.2%)	2 (4.3%)	28 (32.9%)
	don’t know	29 (11.0%; CI: 7.5%–15.5%)	15 (11.4%)	11 (23.9%)	3 (3.5%)
	Missing	1	1	0	0
**PEOPLE can get…**					
treatment against tuberculosis in Bhutan	Yes	231 (87.8%; CI: 83.4%–91.4%)	114 (85.7%)	33 (73.3%)	84 (98.8%)
	No	15 (5.7%; CI: 3.3%–9.3%)	14 (10.5%)	0 (0.0%)	1 (1.2%)
	don’t know	17 (6.5%; CI: 3.8%–10.2%)	5 (3.8%)	12 (26.7%)	0 (0.0%)
	Missing	1	0	1	0
a vaccine against tuberculosis in Bhutan	Yes	124 (47.1%; CI: 40.9%–53.4%)	34 (25.6%)	29 (64.4%)	61 (71.8%)
	No	21 (8.0%; CI: 5.0%–12.1%)	18 (13.5%)	0 (0.0%)	3 (3.5%)
	don’t know	118 (44.9%; CI: 38.7%–51.2%)	81 (60.9%)	16 (35.6%)	21 (24.7%)
	Missing	1	0	1	0
**Vaccination can protect people against bovine tuberculosis**	Yes	143 (54.2%; CI: 48.0%–60.3%)	72 (54.1%)	31 (67.4%)	40 (47.1%)
	No	41 (15.5%; CI: 11.3%–20.6%)	11 (8.3%)	2 (4.3%)	28 (32.9%)
	don’t know	80 (30.3%; CI: 24.9%–36.2%)	50 (37.6%)	13 (28.3%)	17 (20.0%)
	Missing	0	0	0	0
**Human tuberculosis is an important public health problem in Bhutan**	Yes	176 (66.9%; CI: 60.9%–72.6%)	64 (48.5%)	37 (80.4%)	75 (88.2%)
	No	31 (11.8%; CI: 8.1%–16.4%)	20 (15.2%)	1 (2.2%)	10 (11.8%)
	don’t know	56 (21.3%; CI: 16.5%–26.9%)	48 (36.4%)	8 (17.4%)	0 (0.0%)
	Missing	1	1	0	0

*Missing responses were absent from the dataset and were excluded from percentage calculations.

** Overall 95% confidence interval estimated using the Wilson score method.

Overall, farmers demonstrated moderate knowledge about human tuberculosis treatment and prevention, with significant gaps in their understanding of disease presentation and vaccination (Table 2). Most farmers (87.8%, 95% CI: 83.4%–91.4%) were aware that treatment was available in Bhutan, and two-thirds (66.9%, 95% CI: 60.9%–72.6%) recognised tuberculosis as a public health problem. However, only 11.4% (95% CI: 7.8%–16.0%) were aware that infected individuals may look healthy.

Knowledge about vaccination was variable: 47.1% (95% CI: 40.9%–53.4%) knew vaccines were available in Bhutan, and 54.2% (95% CI: 48.0%–60.3%) guessed vaccination could protect against bovine tuberculosis. The stratification of the sample by dzongkhag suggests there are knowledge gradients, with farmers in more remote areas (Haa) showing consistently lower awareness across multiple domains compared to those closer to the capital (Thimphu), particularly regarding vaccine availability (25.6% vs 71.8%) and recognition of tuberculosis as a public health priority (48.5% vs 88.2%).

### Attitudes towards bTB

#### Transmission routes bTB to humans.

Farmers’ risk perceptions varied across different zoonotic transmission routes (Table 3). For dairy consumption, perceptions were evenly distributed: 32.8% (95% CI: 27.2%–38.8%) perceived low risk from consuming milk, while 35.5% (95% CI: 29.8%–41.6%) and 31.7% (95% CI: 26.1%–37.7%) perceived medium and high risk, respectively. About half of all farmers interviewed (47.1%, 95% CI: 40.9%–53.4%) perceived medium risk from consuming cheese, butter, and yogurt.

**Table 3:** Perceptions of zoonotic bovine tuberculosis infection risk among Bhutanese farmers.

Question	Risk Level	Overall (frequency, 95% CI)**	Haa	Paro	Thimphu
**In Bhutan, what do you think is the risk of people getting infected with bovine tuberculosis from…**					
Consuming milk?	Low	86 (32.8%; CI: 27.2%–38.8%)	46 (34.8%)	14 (30.4%)	26 (31.0%)
	Medium	93 (35.5%; CI: 29.8%–41.6%)	49 (37.1%)	18 (39.1%)	26 (31.0%)
	High	83 (31.7%; CI: 26.1%–37.7%)	37 (28.0%)	14 (30.4%)	32 (38.1%)
	Missing*	2	1	0	1
Consuming dairy product (cheese, butter, yogurt)	Low	71 (27.0%; CI: 21.7%–32.8%)	31 (23.3%)	14 (30.4%)	26 (31.0%)
	Medium	124 (47.1%; CI: 40.9%–53.4%)	74 (55.6%)	22 (47.8%)	28 (33.3%)
	High	68 (25.9%; CI: 20.6%–31.7%)	28 (21.1%)	10 (21.7%)	30 (35.7%)
	Missing*	1	0	0	1
Consuming meat?	Low	37 (14.1%; CI: 10.1%–19.0%)	12 (9.0%)	10 (21.7%)	15 (18.1%)
	Medium	79 (30.2%; CI: 24.7%–36.2%)	40 (30.1%)	16 (34.8%)	23 (27.7%)
	High	146 (55.7%; CI: 49.5%–61.8%)	81 (60.9%)	20 (43.5%)	45 (54.2%)
	Missing*	2	0	0	2
Consuming an animal with signs of btB?	Low	15 (5.7%; CI: 3.3%–9.3%)	10 (7.5%)	1 (2.2%)	4 (4.8%)
	Medium	41 (15.6%; CI: 11.3%–20.8%)	34 (25.6%)	2 (4.3%)	5 (6.0%)
	High	207 (78.7%; CI: 73.2%–83.6%)	89 (66.9%)	43 (93.5%)	75 (89.3%)
	Missing*	1	0	0	1
Contact with animals?	Low	45 (17.1%; CI: 12.7%–22.4%)	20 (15.0%)	9 (19.6%)	16 (19.0%)
	Medium	121 (46.0%; CI: 39.8%–52.3%)	76 (57.1%)	27 (58.7%)	18 (21.4%)
	High	97 (36.9%; CI: 31.0%–43.1%)	37 (27.8%)	10 (21.7%)	50 (59.5%)
	Missing*	1	0	0	1
Contact with people?	Low	62 (23.6%; CI: 18.5%–29.3%)	31 (23.3%)	18 (39.1%)	13 (15.5%)
	Medium	104 (39.5%; CI: 33.5%–45.8%)	54 (40.6%)	25 (54.3%)	25 (29.8%)
	High	97 (36.9%; CI: 31.0%–43.1%)	48 (36.1%)	3 (6.5%)	46 (54.8%)
	Missing*	1	0	0	1
allowing cattle into your dwelling/living area?	Low	28 (10.7%; CI: 7.2%–15.2%)	9 (6.8%)	10 (21.7%)	9 (10.8%)
	Medium	93 (35.5%; CI: 29.8%–41.6%)	41 (30.8%)	27 (58.7%)	25 (30.1%)
	High	141 (53.8%; CI: 47.6%–59.9%)	83 (62.4%)	9 (19.6%)	49 (59.0%)
	Missing*	2	0	0	2
sharing a water source with your cattle?	Low	40 (15.3%; CI: 11.1%–20.5%	21 (15.9%)	8 (17.4%)	11 (13.3%)
	Medium	113 (43.3%; CI: 37.1%–49.6%)	71 (53.8%)	26 (56.5%)	16 (19.3%)
	High	108 (41.4%; CI: 35.2%–47.8%)	40 (30.3%)	12 (26.1%)	56 (67.5%)
	Missing*	1	1	0	2
**What do you think is the risk of infection from bovine tuberculosis among…**					
Dairy farmers	Low	48 (18.2%; CI: 13.7%–23.4%)	25 (18.8%)	8 (17.4%)	15 (17.6%)
	Medium	55 (20.8%; CI: 16.0%–26.4%)	29 (21.8%)	17 (37.0%)	9 (10.6%)
	High	161 (61.0%; CI: 54.8%–66.9%)	79 (59.4%)	21 (45.7%)	61 (71.8%)
Cattle traders	Low	21 (8.0%; CI: 5.0%–12.1%)	10 (7.5%)	1 (2.2%)	10 (11.8%)
	Medium	107 (40.5%; CI: 34.5%–46.7%)	80 (60.2%)	11 (23.9%)	16 (18.8%)
	High	136 (51.5%; CI: 45.3%–57.7%)	43 (32.3%)	34 (73.9%)	59 (69.4%)
Dairy consumers	Low	(12.5%; CI: 8.7%–17.2%)	10 (7.5%)	9 (19.6%)	14 (16.5%)
	Medium	117 (44.3%; CI: 38.2%–50.6%)	62 (46.6%)	19 (41.3%)	36 (42.4%)
	High	114 (43.2%; CI: 37.1%–49.4%)	61 (45.9%)	18 (39.1%)	35 (41.2%)
Meat consumers	Low	18 (6.8%; CI: 4.1%–10.6%)	3 (2.3%)	9 (19.6%)	6 (7.1%)
	Medium	68 (25.8%; CI: 20.5%–31.7%)	23 (17.3%)	15 (32.6%)	30 (35.3%)
	High	178 (67.4%; CI: 61.4%–73.1%)	107 (80.5%)	22 (47.8%)	49 (57.6%)
Veterinarians/Paraveterinarians	Low	125 (47.3%; CI: 41.1%–53.6%)	56 (42.1%)	32 (69.6%)	37 (43.5%)
	Medium	68 (25.8%; CI: 20.5%–31.7%)	35 (26.3%)	5 (10.9%)	28 (32.9%)
	High	71 (26.9%; CI: 21.5%–32.9%)	42 (31.6%)	9 (19.6%)	20 (23.5%)

*Missing responses were absent from the dataset and were excluded from percentage calculations.

** Overall 95% confidence interval estimated using the Wilson score method.

Meat consumption was perceived as significantly riskier: more than half of the farmers (55.7%, 95% CI: 49.5%–61.8%) regarded it as high risk, and only 14.1% (95% CI: 10.1%–19.0%) saw it as low risk. This concern was strongest in Haa (60.9% high risk), compared to Paro (43.5%) and Thimphu (54.2%). The perception of risk increased sharply when it involved animals showing signs of bTB, with over three-quarters of farmers (78.7%, 95% CI: 73.2%–83.6%) viewing this as high risk. Regarding animal contact, farmers were divided: 46.0% (95% CI: 39.8%–52.3%) perceived medium risk from direct animal contact, while 36.9% (95% CI: 31.0%–43.1%) saw it as high risk. Geographic differences were significant, with Thimphu farmers showing markedly higher concern about infection through direct contact with animals (59.5% high risk) compared to Haa (27.8%) and Paro (21.7%). Human-to-human transmission was perceived as a lower risk overall, with 39.5% (95% CI: 33.5%–45.8%) rating it as medium risk, although concern was again higher among Thimphu farmers than in other locations.

Questions about housing and water-sharing practices showed that over half of farmers (53.8%, 95% CI: 47.6%–59.9%) saw allowing cattle into living areas as high risk, with Haa (62.4%) and Thimphu (59.0%) farmers expressing more concern than Paro farmers (19.6%). Sharing water sources with cattle was seen as medium (43.3%, 95% CI: 37.1%–49.6%) to high risk (41.4%, 95% CI: 35.2%–47.8%) by most farmers, with Thimphu farmers showing the greatest concern (67.5% high risk) compared to Haa (30.3%) and Paro (26.1%). Detailed frequencies are shown in Table 3.

#### Occupational risk of bTB infection.

Farmers perceived themselves as having substantial infection risk, with 61.0% (95% CI: 54.8%–66.9%) rating dairy farmers as high risk and only 18.2% (95% CI: 13.7%–23.4%) as low risk. This self-perception was relatively consistent across dzongkhags, with Thimphu farmers showing elevated concern (71.8% high risk vs. 59.4% in Haa and 45.7% in Paro). Farmers also perceived cattle traders as a group with high occupational risk, with 51.5% (95% CI: 45.3%–57.7%) considering them high risk and only 8.0% (95% CI: 5.0%–12.1%) as low risk.

Farmers’ perception of bTB risk among dairy consumers was evenly split between medium (44.3%, 95% CI: 38.2%–50.6%) and high risk (43.2%, 95% CI: 37.1%–49.4%), with relatively consistent patterns across dzongkhags. In contrast, meat consumers were perceived by farmers as having substantially higher risk (67.4%, 95% CI: 61.4%–73.1%), and only 6.8% (95% CI: 4.1%–10.6%) rated them as low risk. This perception was most pronounced among Haa farmers (80.5% high risk) than those in Thimphu (57.6%) and Paro (47.8%), aligned with meat consumption perceptions reported earlier. Farmers perceived veterinarians and paraveterinarians as having the lowest risk (47.3% 95% CI: 41.1%–53.6%) among milk value chain stakeholders.

#### Transmission routes for bTB infection in cattle.

Over three-quarters of farmers perceived introducing new cattle into herds as a medium to high risk of bTB infection for their cattle, while only 23.1% (95% CI: 18.2%–28.7%) considered it low risk. Similarly, nearly 90% of farmers saw contact with neighbouring herds as a medium to high risk of cattle-to-cattle bTB transmission. Thimphu farmers showed the highest concern, with 43.5% and 45.9% perceiving it as high risk, respectively, whereas Haa farmers expressed more moderate views. In contrast, contact with veterinary professionals was consistently seen as the safest interaction for their cattle, with only 17.8% (95% CI: 13.3%–23.2%) considering it high risk. Wildlife and other livestock contacts were generally perceived as a moderate risk of bTB infection across all farmers. Contact with dogs emerged as the lowest overall risk, with 43.7% (95% CI: 37.6%–50.0%). Detailed results are presented in table 4. Farmers were also asked about their perceptions of various strategies to reduce cow-to-cow bTB transmission. Vaccination and isolation were perceived as the two most effective interventions, each receiving over 40% of mentions; however, about 15% of farmers indicated they did not know how the disease could be mitigated (Appendix B in S1 Text).

**Table 4:** Perceptions of animal-to-animal bovine tuberculosis infection risk among Bhutanese farmers.

Question	Risk Level	Overall (frequency, 95% CI)**	Haa	Paro	Thimphu
**In Bhutan, what do you think is the risk of cattle getting infected with bovine tuberculosis when…**					
buying new cattle into your herd?	Low	61 (23.1%; CI: 18.2%–28.7%)	36 (27.1%)	3 (6.5%)	22 (25.9%)
	Medium	108 (40.9%; CI: 34.9%–47.1%)	53 (39.8%)	29 (63.0%)	26 (30.6%)
	High	95 (36.0%; CI: 30.2%–42.1%)	44 (33.1%)	14 (30.4%)	37 (43.5%)
	Missing	0	0	0	0
contact between your cattle and neighbouring herds?	Low	35 (13.3%; CI: 9.4%–18.1%)	21 (15.8%)	2 (4.3%)	12 (14.1%)
	Medium	143 (54.2%; CI: 48.0%–60.3%)	83 (62.4%)	26 (56.5%)	34 (40.0%)
	High	86 (32.6%; CI: 26.9%–38.7%)	29 (21.8%)	18 (39.1%)	39 (45.9%)
	Missing	0	0	0	0
contact between your cattle and veterinarians/paravets?	Low	85 (32.2%; CI: 26.6%–38.2%)	41 (30.8%)	19 (41.3%)	25 (29.4%)
	Medium	132 (50.0%; CI: 43.8%–56.2%)	66 (49.6%)	21 (45.7%)	45 (52.9%)
	High	47 (17.8%; CI: 13.3%–23.2%)	26 (19.5%)	6 (13.0%)	15 (17.6%)
	Missing	0	0	0	0
there is contact between your cattle and wildlife?	Low	68 (25.8%; CI: 20.5%–31.7%)	32 (24.1%)	17 (37.0%)	19 (22.4%)
	Medium	124 (47.0%; CI: 40.8%–53.2%)	66 (49.6%)	16 (34.8%)	42 (49.4%)
	High	72 (27.3%; CI: 21.9%–33.3%)	35 (26.3%)	13 (28.3%)	24 (28.2%)
	Missing	0	0	0	0
contact between your cattle and other livestock *spp*.?	Low	83 (31.6%; CI: 26.0%–37.6%)	50 (37.9%)	13 (28.3%)	20 (23.5%)
	Medium	114 (43.3%; CI: 37.2%–49.6%)	55 (41.7%)	19 (41.3%)	40 (47.1%)
	High	66 (25.1%; CI: 19.9%–30.9%)	27 (20.5%)	14 (30.4%)	25 (29.4%)
	Missing*	1	0	0	1
there is contact between your cattle and dogs?	Low	115 (43.7%; CI: 37.6%–50.0%)	73 (54.9%)	19 (42.2%)	23 (27.1%)
	Medium	85 (32.3%; CI: 26.7%–38.4%)	41 (30.8%)	15 (33.3%)	29 (34.1%)
	High	63 (24.0%; CI: 18.8%–29.8%)	19 (14.3%)	11 (24.4%)	33 (38.8%)
	Missing*	1	0	1	0

* Missing responses were absent from the dataset and were excluded from percentage calculations within each dzongkhag.

** Overall 95% confidence interval estimated using the Wilson score method.

### Practices related to bTB

#### Milk preferences.

Nearly half of all farmers (44.1%) consumed potentially unsafe milk either through raw intake (5.7%, 95% CI: 3.2%–9.4%) or by possibly “inadequate boiling” of less than 10 minutes (38.4%, 95% CI: 32.2%–44.8%). The remaining farmers practised safe consumption by adequately boiling milk for more than 10 minutes (34.3%, 95% CI: 28.3%–40.7%), using commercial pasteurised milk (2.0%, 95% CI: 0.7%–4.6%), or milk powder (17.1%, 95% CI: 12.6%–22.5%). Haa farmers reported the most concerning practices, with 64.9% consuming potentially unsafe milk, compared to only about 20% of farmers in Thimphu and Paro, many of whom reported consuming milk powder (Table 5).

**Table 5:** Milk consumption preferences among Bhutanese farmers.

What type of milk do you consume most often?	Overall (frequency, 95% CI)**	Haa	Paro	Thimphu
Raw/fresh	14 (5.7%; CI: 3.2%–9.4%)	13 (9.8%)	0 (0.0%)	1 (1.2%)
Boiled (less than 10mins)	94 (38.4%; CI: 32.2%–44.8%)	72 (54.1%)	6 (21.4%)	16 (19.0%)
Boiled (more than 10 mins)	84 (34.3%; CI: 28.3%–40.7%)	47 (35.3%)	10 (35.7%)	27 (32.1%)
Pasteurised milk in tetra pack	5 (2.0%; CI: 0.7%–4.6%)	1 (0.8%)	1 (3.6%)	3 (3.6%)
Milk powder	42 (17.1%; CI: 12.6%–22.5%)	0 (0.0%)	8 (28.6%)	34 (40.5%)
I don’t consume milk	6 (2.4%; CI: 0.9%–5.2%)	0 (0.0%)	3 (10.7%)	3 (3.6%)
Missing*	19	0	18	1

* Missing responses were absent from the dataset and were excluded from percentage calculations within each dzongkhag.

** Overall 95% confidence interval estimated using the Wilson score method.

#### Cattle-sourced products.

Consumption frequencies were defined as always (daily), frequently (at least weekly), rarely (monthly or less), and never. Dairy products and milk dominated the consumption patterns reported: 79.8% (95% CI: 74.4%–84.6%) and 66.5% (95% CI: 60.2%–72.4%), respectively, always consumed them. Meat consumption was moderate, with 53.9% (95% CI: 47.7%–60.0%) consuming it frequently and 39.5% (95% CI: 33.6%–45.7%) only rarely. Home production was the primary source for dairy products: 86.9% (95% CI: 82.1%–90.8%) produced their milk, and 77.7% (95% CI: 72.2%–82.6%) sourced dairy products from their own production, with little reliance on friends, family, or neighbours. Conversely, meat is mainly sourced from the market at 90.3% (95% CI: 86.0%–93.6%), with minimal home production (3.1%, 95% CI: 1.4%–6.0%) or sourcing from friends, family, and neighbours (3.9%, 95% CI: 1.9%–7.1%) (Table 6).

**Table 6:** Bhutanese farmers’ frequency of consumption and source of animal-sourced products.

Questions	Frequency	Overall (frequency, 95% CI)**	Haa	Paro	Thimphu
**How often do you consume…**					
Milk	Always	163 (66.5%; CI: 60.2%–72.4%)	128 (96.2%)	3 (10.7%)	32 (38.1%)
	Frequently	30 (12.2%; CI: 8.4%–17.0%)	5 (3.8%)	8 (28.6%)	17 (20.2%)
	Rarely	46 (18.8%; CI: 14.1%–24.3%)	0 (0.0%)	14 (50.0%)	32 (38.1%)
	Never	6 (2.4%; CI: 0.9%–5.2%)	0 (0.0%)	3 (10.7%)	3 (3.6%)
	Missing	19	0	18	1
Dairy products (cheese, yogurt, butter)	Always	210 (79.8%; CI: 74.4%–84.6%)	126 (95.5%)	26 (56.5%)	58 (68.2%)
	Frequently	37 (14.1%; CI: 10.1%–19.0%)	6 (4.5%)	16 (34.8%)	15 (17.6%)
	Rarely	15 (5.7%; CI: 3.3%–9.3%)	0 (0.0%)	3 (6.5%)	12 (14.1%)
	Never	1 (0.4%; CI: 0.0%–2.1%)	0 (0.0%)	1 (2.2%)	0 (0.0%)
	Missing	1	1	0	0
Meat	Always	10 (3.9%; CI: 1.9%–7.0%)	7 (5.3%)	0 (0.0%)	3 (3.7%)
	Frequently	139 (53.9%; CI: 47.7%–60.0%)	113 (85.0%)	21 (48.8%)	5 (6.1%)
	Rarely	102 (39.5%; CI: 33.6%–45.7%)	13 (9.8%)	19 (44.2%)	70 (85.4%)
	Never	7 (2.7%; CI: 1.1%–5.5%)	0 (0.0%)	3 (7.0%)	4 (4.9%)
	Missing	6	0	3	3
**Where do you source your…**					
Milk	Market	24 (9.8%; CI: 6.4%–14.3%)	0 (0.0%)	2 (7.1%)	22 (26.2%)
	Homemade	213 (86.9%; CI: 82.1%–90.8%)	133 (100.0%)	21 (75.0%)	59 (70.2%)
	Friend/family/neighbour	2 (0.8%; CI: 0.1%–2.9%)	0 (0.0%)	2 (7.1%)	0 (0.0%)
	Don’t consume	6 (2.4%; CI: 0.9%–5.2%)	0 (0.0%)	3 (10.7%)	3 (3.6%)
	Missing	19	0	18	1
Dairy products (cheese, butter, yogurt)	Market	52 (19.7%; CI: 15.0%–25.1%)	0 (0.0%)	21 (45.7%)	31 (36.5%)
	Homemade	205 (77.7%; CI: 72.2%–82.6%)	133 (100.0%)	18 (39.1%)	54 (63.5%)
	Friend/family/neighbour	6 (2.3%; CI: 0.8%–4.9%)	0 (0.0%)	6 (13.0%)	0 (0.0%)
	Don’t consume	1 (0.4%; CI: 0.0%–2.1%)	0 (0.0%)	1 (2.2%)	0 (0.0%)
	Missing	0	0	0	0
Meat	Market	232 (90.3%; CI: 86.0%–93.6%)	115 (86.5%)	40 (93.0%)	77 (95.1%)
	Homemade	8 (3.1%; CI: 1.4%–6.0%)	8 (6.0%)	0 (0.0%)	0 (0.0%)
	Friend/family/neighbour	10 (3.9%; CI: 1.9%–7.1%)	10 (7.5%)	0 (0.0%)	0 (0.0%)
	Don’t consume	7 (2.7%; CI: 1.1%–5.5%)	0 (0.0%)	3 (7.0%)	4 (4.9%)
	Missing	7	0	3	4

*Missing responses were absent from the dataset and were excluded from percentage calculations within each dzongkhag.

** Overall 95% confidence interval estimated using the Wilson score method.

Overall, Haa farmers showed the highest dairy consumption (96.2% always consuming milk and 95.5% always consuming dairy products) with exclusive reliance on home production. Thimphu farmers had diverse consumption habits and depended more on the market, with 26.2% for milk and 36.5% for dairy products. Regarding meat, sourcing patterns were relatively similar across the locations, although Haa farmers engaged more in home production and local sourcing (Table 6).

#### Farming practices.

Farmers reported high compliance with hand hygiene practices: 66.7% (95% CI: 60.7%–72.3%) and 89.8% (95% CI: 85.4%–93.2%) of farmers always washed their hands after cattle contact and before and after milking, respectively. However, the use of protective equipment (PPE) was poor, with only 20.8% (95% CI: 16.0%–26.4%) reporting always wearing PPE when treating cattle and 18.6% (95% CI: 14.0%–24.0%) when handling birth material. One-third of the farmers interviewed never used PPE for cattle treatment or handling of birth materials. Milking equipment sanitation was always practised by 59.5% (95% CI: 53.3%–65.5%), and only 4.2% (95% CI: 2.1%–7.4%) never did it. Farm sanitation was uncommon, with only 12.5% (95% CI: 8.7%–17.2%) always cleaning farms and 32.7% (95% CI: 27.0%–38.9%) never doing it (Table 7).

**Table 7:** Milking, preventive, and cleaning practices among Bhutanese farmers.

Questions	Frequency	Overall (frequency, 95% CI)**	Haa	Paro	Thimphu
**How often do you…**					
Wash your hands after contact with cattle	Always	176 (66.7%; CI: 60.7%–72.3%)	55 (41.4%)	44 (95.7%)	77 (90.6%)
	Frequently	81 (30.7%; CI: 25.1%–36.7%)	75 (56.4%)	1 (2.2%)	5 (5.9%)
	Rarely	(2.7%; CI: 1.1%–5.5%)	3 (2.3%)	1 (2.2%)	3 (3.5%)
	Never	0 (0.0%; CI: 0.0%–1.4%)	0 (0.0%)	0 (0.0%)	0 (0.0%)
wash your hands before and after milking cattle	Always	237 (89.8%; CI: 85.4%–93.2%)	111 (83.5%)	46 (100.0%)	80 (94.1%)
	Frequently	27 (10.2%; CI: 6.8%–14.6%)	22 (16.5%)	0 (0.0%)	5 (5.9%)
	Rarely	0 (0.0%; CI: 0.0%–1.4%)	0 (0.0%)	0 (0.0%)	0 (0.0%)
	Never	0 (0.0%; CI: 0.0%–1.4%)	0 (0.0%)	0 (0.0%)	0 (0.0%)
Wear PPE when treating cattle	Always	55 (20.8%; CI: 16.0%–26.4%)	16 (12.0%)	8 (17.4%)	31 (36.5%)
	Frequently	34 (12.9%; CI: 9.1%–17.7%)	23 (17.3%)	7 (15.2%)	4 (4.7%)
	Rarely	100 (37.9%; CI: 31.9%–44.1%)	54 (40.6%)	26 (56.5%)	20 (23.5%)
	Never	75 (28.4%; CI: 23.0%–34.4%)	40 (30.1%)	5 (10.9%)	30 (35.3%)
Wear PPE when handling birth material	Always	49 (18.6%; CI: 14.0%–24.0%)	1 (0.8%)	9 (19.6%)	39 (45.9%)
	Frequently	16 (6.1%; CI: 3.5%–9.7%)	7 (5.3%)	4 (8.7%)	5 (5.9%)
	Rarely	101 (38.3%; CI: 32.3%–44.6%)	62 (46.6%)	28 (60.9%)	11 (12.9%)
	Never	98 (37.1%; CI: 31.2%–43.4%)	63 (47.4%)	5 (10.9%)	30 (35.3%)
Clean and sanitise milking tools before and after?	Always	157 (59.5%; CI: 53.3%–65.5%)	74 (55.6%)	31 (67.4%)	52 (61.2%)
	Frequently	77 (29.2%; CI: 23.7%–35.2%)	51 (38.3%)	4 (8.7%)	22 (25.9%)
	Rarely	19 (7.2%; CI: 4.4%–11.0%)	4 (3.0%)	11 (23.9%)	4 (4.7%)
	Never	11 (4.2%; CI: 2.1%–7.4%)	4 (3.0%)	0 (0.0%)	7 (8.2%)
Clean and sanitise the farm?	Always	33 (12.5%; CI: 8.7%–17.2%)	4 (3.0%)	5 (10.9%)	24 (28.6%)
	Frequently	42 (16.0%; CI: 11.7%–21.2%)	24 (18.0%)	8 (17.4%)	10 (11.9%)
	Rarely	102 (38.8%; CI: 32.8%–45.0%)	56 (42.1%)	16 (34.8%)	30 (35.7%)
	Never	86 (32.7%; CI: 27.0%–38.9%)	49 (36.8%)	17 (37.0%)	20 (23.8%)

*Missing responses were absent from the dataset and were excluded from percentage calculations within each dzongkhag.

** Overall, 95% confidence interval estimated using the Wilson score method.

Thimphu farmers reported superior practices across most domains, with 90.6% always washing hands after cattle contact and 45.9% always using PPE for birth material handling. Paro farmers reported excellent hand hygiene (95.7% and 100% for the two hand-washing practices) but moderate PPE use. Haa farmers reported the poorest hygiene practices, with only 41.4% always washing hands after cattle contact and minimal PPE use (12.0% for cattle treatment, 0.8% for birth material) (Table 7).

#### Self-reported health-care practices and veterinary service use.

Most farmers (81.4%, 95% CI: 76.2-85.9%) reported receiving at least one medical check-up annually, with higher rates observed in Haa (88.2%). Self-reported tuberculosis testing history was limited, with only 8.7% (95% CI: 5.6-12.9%) of farmers reporting ever being tested, with a gradient of regional variation: Thimphu (17.6%), Paro (13.0%), and Haa (1.5%). Self-reported treatment history for tuberculosis was reported by 4.9% (95% CI: 2.7-8.2%) of all farmers. Tuberculosis vaccination status showed considerable uncertainty, with 8.0% (95% CI: 5.0-12.1%) reporting vaccination, 72.7% reporting no vaccination, and 19.3% uncertain of their status. Haa farmers reported the lowest vaccination rate (99.2% not vaccinated) across dzongkhags (Appendix B in S1 Text)

Veterinary services were the primary source of cattle healthcare for 84.8% (95% CI: 79.8-88.9%) of the farmers interviewed, though this varied by region: Haa (100.0%), Thimphu (83.5%), and Paro (43.5%). In Paro, alternative care sources were common, with equal reliance on veterinary services or friends, family and neighbours (43.5% each). Self-treatment of animals was reported by only 4.9% of the farmers. Reporting cattle diseases to the authorities was deemed easy or very easy by 91.3% (95% CI: 87.2-94.4%) of the farmers. Similarly, 85.6% (95% CI: 80.8-89.6%) found obtaining veterinary assistance for sick animals easy or very easy. However, Paro and Thimphu farmers reported more difficulty (23.9% and 31.8% rating it as hard, respectively) compared to those in Haa (0.0%). Details are shown in Appendix B in S1 Text.

### Dimensional reduction and clustering

**Fig 2:**
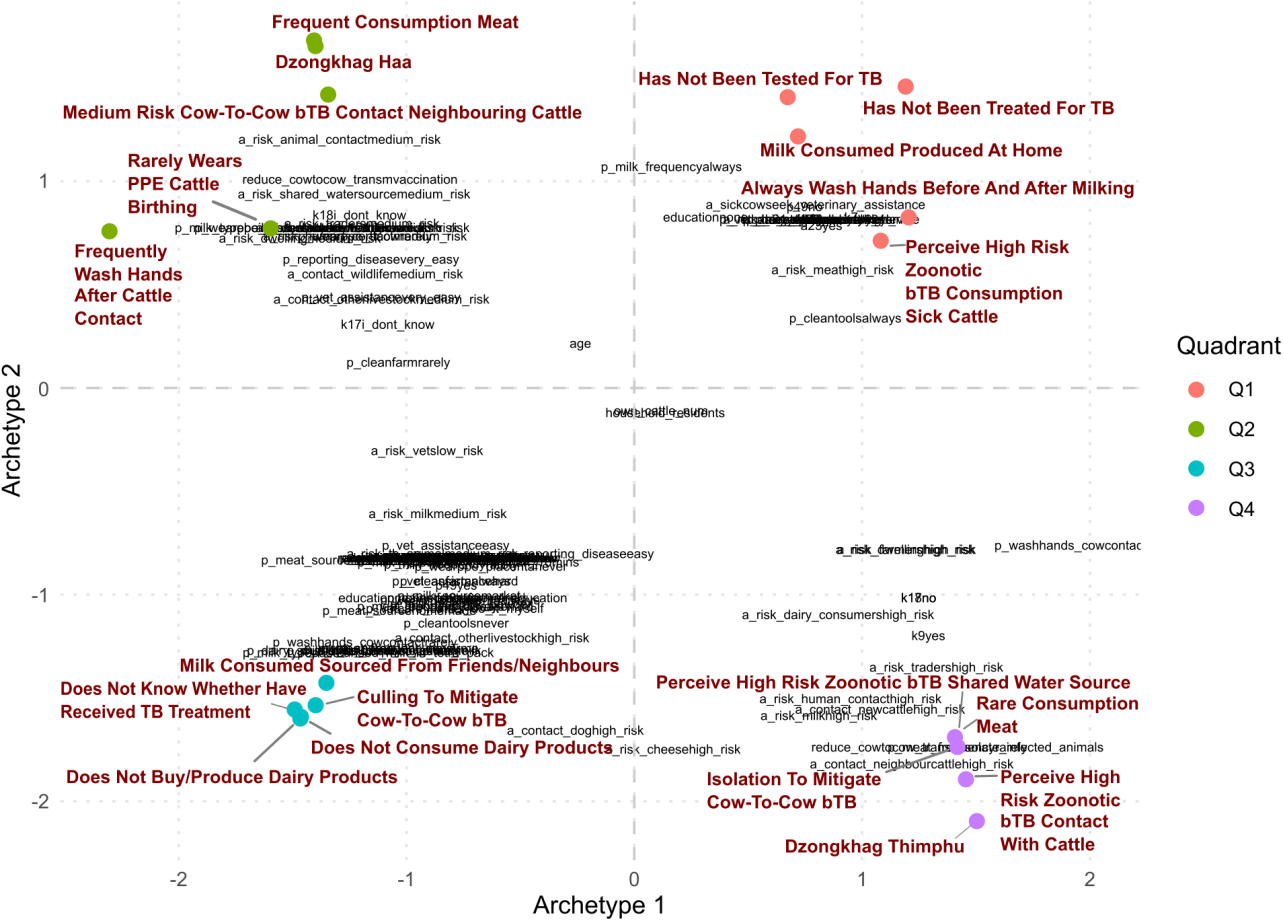
Projection of variables onto two archetypal dimensions. The top five extreme values in each quadrant are highlighted.

The GLRM matrix factorisation resulted in a two-archetype solution, with archetypes 1 and 2 explaining 79.5% and 20.5% of the total data variation, respectively (Appendix C in S1 Text). The top three variables with high unilateral loads on Archetype 1 were “always washing hands after contact with cattle”, “having Thimphu as dzongkhag of residence”, and “perceived high risk of zoonotic bTB due to contact with cattle”. For Archetype 2, “frequent meat consumption”, “having Haa as dzongkhag of residence”, and “not having received treatment for tuberculosis” were the top three variables. The projection of variables onto two archetypal dimensions shows specific clustering patterns of practice and attitude associated with the dzongkhag of residence (Fig 2): quadrant 2 (top left) shows that dzongkhag Haa is associated with lower perceptions of risk, lower biosecurity practices and higher consumption of meat; in contrast, dzongkhag Thimphu (quadrant 4), exhibits an association with high-risk-perception factors, particularly concerning the risk of zoonotic bTB (contact with animals and sharing a water source), rare consumption of meat and mitigation of cow-to-cow bTB transmission by animal isolation.

K-means clustering resulted in a two-cluster or three-cluster split as an optimal solution to describe data patterns (Table 8). We discarded the three-cluster split as the third cluster was only characterised by variables with high unilateral loads on archetype 2 (i.e., variables with loads equal to or above 1 for archetype 2, and equal or lower than -1 for archetype 1), which provided limited insight into data variation (details in Appendix C in S1 Text). The two-cluster solution aligned with the patterns observed using the archetypal projection (Fig 2) and characterised using the top five variables with high unilateral loads on Archetype 1 (which explains most of the data variation): dzongkhag, washing hands after contact with cattle, perception of zoonotic bTB risk associated with animal contact, perceived best method to reduce cow-to-cow bTB transmission, and frequency of meat consumption (Table 2). Cluster 1 included 83 of the 85 farmers with residence in Thimphu, who reported a high-risk perception of zoonotic bTB due to contact with cattle and high rates of handwashing after being in touch with them. Consistent with this, further exploration of factors with high unilateral loads on Archetype 1, shows that individuals in this cluster perceive occupational contact (farmer, trader), as well as environmental contact with cattle (water source, dwelling) as high-risk of zoonotic bTB (Appendix C in S1 Text).

**Table 8:** Characteristics of two-cluster K-means solution using the top five variables with high loads on Archetype 1.

Characteristic n (%)	Cluster 1 n = 134	Cluster 2 n = 130
**Dzongkhag**		
Haa	28 (20.9%)	105 (80.8%)
Paro	23 (17.2%)	23 (17.7%)
Thimphu	83 (61.9%)	2 (1.5%)
**Washing hands after contact with cattle**		
Always	121 (90.3%)	55 (42.3%)
Frequently	11 (8.2%)	70 (53.8%)
Rarely	2 (1.5%)	5 (3.8%)
Never	0 (0.0%)	0 (0.0%)
**Risk zoonotic bTB cattle contact**		
High risk	83 (62.4%)	14 (10.8%)
Medium risk	28 (21.1%)	93 (71.5%)
Low risk	22 (16.5%)	23 (17.7%)
Never	0 (0.0%)	0 (0.0%)
Missing*	1	0
**Reduce cow-to-cow bTB transmission**		
Vaccination	27 (20.1%)	88 (67.7%)
Tuberculin skin test	0 (0.0%)	0 (0.0%)
Isolate infected animals	90 (67.2%)	19 (14.6%)
Culling	1 (0.7%)	1 (0.8%)
I don’t know	16 (11.9%)	22 (16.9%)
**Frequency meat consumption**		
Always	6 (4.7%)	4 (3.1%)
Frequently	30 (23.4%)	109 (83.8%)
Rarely	86 (67.2%)	16 (12.3%)
Never	6 (4.7%)	1 (0.8%)
Missing*	6	0

*Missing responses were absent from the dataset and were excluded from percentage calculations within each dzongkhag.

#### Correlations of risk perception.

Farmers, on average, perceive bTB risk as medium for both animals and humans. The human risk index is slightly higher than the animal risk index in all locations. The most significant gap between index scores was in Haa (0.35) and the smallest in Paro (0.08). The highest average risk index scores occurred in Thimphu, and the lowest in Haa. The Pearson correlation coefficient showed that human and animal risk indices are overall correlated across all locations (Pearson correlation: 0.39); However, this correlation is primarily driven by the dzongkhag of Haa (Pearson correlation: 0.51). The Pearson coefficients for Thimphu and Paro highlighted no difference between their animal and human bTB risk perceptions (Table 9).

**Table 9:** Average risk indices and correlation coefficients across dzongkhags.

	Haa	Paro	Thimphu	All locations
Animal risk index	1.90	2.02	2.10	1.99
Human risk index	2.25	2.10	2.40	2.27
Pearson correlation	0.51^***^	0.36	0.25	0.39^*^

“Significant at the 1% level.

## Discussion

We found a near-universal lack of bTB awareness among dairy farmers supplying milk and dairy products to the capital of Bhutan, as well as variations in risk perception and practices across dzongkhags. The lack of bTB knowledge is consistent with observations reported in other LMICs [17]; however, the implications for Bhutan are particularly concerning due to its substantial nutritional and economic reliance on dairy farming and the close human-animal interactions involved in traditional farming practices. Only 1.9% of farmers recognised the zoonotic potential of bTB, indicating that even among the small minority with some disease awareness, understanding of human health implications of bTB was virtually absent. The knowledge disconnect between human and animal tuberculosis highlights why bTB threatens public health, remains neglected and is difficult to mitigate in low socio-economic agrarian settings with limited access to pasteurisation [1,3]. The contribution of bTB to the burden of human tuberculosis remains largely unquantified in the region [13], and Bhutan reports a high incidence of extra-pulmonary human tuberculosis [34,35], which is frequently associated with zoonotic tuberculosis infection in the clinical literature [3]. Variations in knowledge, attitudes, and practices towards bTB and other zoonoses have been documented in different contexts [36] highlighting that a holistic, effective One Health management of bTB must encompass socio-economic, cultural, political, and religious dimensions beyond pathogen-centric approaches [37].

The general lack of awareness of bTB and its zoonotic potential in our study is unsurprising, as no veterinary or public health activities have ever been conducted in Bhutan specifically targeting bTB. However, it represents a significant problem because *M. bovis* is present in Bhutan [12], is prevalent in neighbouring countries [15,38,39], and 79.8% of farmers in our study consume dairy products daily, and 86.9% rely on home production, creating substantial vulnerability for zoonotic transmission. Moreover, the presence of bTB in Bhutan and the region negatively affects cattle health and well-being, diminishing fertility, milk, and overall productivity, ultimately impacting farmers’ livelihoods. Milk production losses due to bTB have been estimated at around 10% [40] and up to 18% in neighbouring Bangladesh [38]. Calving rates could be reduced by approximately 5%, affecting fertility and herd composition [40]. Like other LMIC settings, awareness of human tuberculosis in our study did not guarantee bTB awareness [17].

Socioeconomic factors and education levels influence tuberculosis knowledge, with better-educated individuals generally more informed [41]. This is consistent with our finding of diminishing gradient of education and bTB knowledge observed from the capital Thimphu towards the remote dzongkhag of Haa (Table 1). Risk perceptions and practices also featured a geographical gradient pattern: farmers in Thimphu had the highest risk-perception index scores, followed by Paro and Haa. Generally, a more significant proportion of farmers in Thimphu (the capital) reported high-risk perceptions of zoonotic bTB infection for different types of product consumption and types of contact, and these were accompanied by practices that demonstrated risk aversion (e.g., always washing hands after contact with cattle). This pattern may stem from farmers’ higher education levels and exposure to veterinary and public health messaging in Thimphu, the capital of Bhutan, versus the more remote dzongkhags.

The relationship between risk perception and behaviour was complex in Haa, where we observed a significant correlation between bTB risk perception for animals and humans (r = 0.51) alongside the largest gap between human and animal risk-perception scores. Paradoxically, despite Haa farmers reporting high risk perceptions for zoonotic bTB associated with milk consumption, nearly 100% consume milk daily, and two-thirds (64.9%) consumed potentially unsafe milk through raw intake or inadequate boiling. The 10-minute boiling threshold in our study accounts for the lack of temperature monitoring in household settings, variable heating methods used by farmers, and ‘boiling’ often referring in Bhutan to heating milk without necessarily maintaining a rolling boil, making it essential to specify a duration long enough to inactivate pathogens [42]. Individual and societal perceptions of food-related health risks are multidimensional [42] and can be influenced by location, age, culture and gender, among others [43–45]. As reflected in our study, most farmers in Haa are women [46], and previous studies have shown that women are more prone to consider food bacteria as a health risk than men [47,48]. We hypothesise that easier access to locally produced milk and meat may result in frequent consumption despite risk concerns, illustrating how convenience can override safety considerations in resource-constrained settings. Finally, the use of PPE and farm disinfection was infrequent across dzongkhags (with a slight downward trend from Thimphu to Haa), consistent with reports of smallholder farmers in the region [49]. The poor adoption of protective equipment despite relatively good hand hygiene compliance (66.7-89.8%) suggests that interventions to mitigate bTB risk and other zoonoses should focus on accessible, culturally appropriate protective measures rather than resource-intensive solutions.

We explored patterns of variation and response gradients using a combination of GLRM and K-means clustering. First, we produced Archetypes to identify factor relationships that determine data variation. Second, we intersected the Archetypes to produce quadrants and show how factors group together based on their relationship with both archetypes simultaneously. Third, we produced clusters to group farmers with common characteristics across the archetypes. We found that grouping factors in the archetype space persisted when farmers were grouped using unsupervised k-means clustering. In practical terms, this suggests that similar practices are shared by farmers living under similar geopolitical conditions (i.e., dzongkhags). Therefore, geographical, social, and infrastructural differences are likely important determinants of attitude and practice. For example, traditional community grasslands in Haa [38] may reduce the risk perception of animals contacting neighbouring cattle. This type of communal production may also explain why these farmers consider vaccination—not available in Bhutan but investigated elsewhere [1]—as the most effective way to mitigate bTB transmission over animal isolation.

This study complements a study of dairy consumers [50], giving insight into husbandry practices, milk production and consumption patterns, and stakeholder relations relevant *to M. bovis*. At the beginning of the survey, we briefed participants about bTB and then explicitly asked whether they had heard of bTB before this briefing, increasing our confidence that the almost complete absence of knowledge among farmers is representative of our target population. However, social desirability bias may have influenced responses regarding hygiene practices and some risk perceptions, potentially leading to overreporting of safer behaviours. We acknowledge that most surveys are unlikely to produce data free of recall bias [51] and therefore, the interpretation of knowledge, attitudes and practices should be cautious. Moreover, this study covers the population of farmers sourcing the milk value chain of Thimphu. Therefore, these results should not be assumed to be immediately representative of the country’s dairy farmer population, as previous studies have shown there are relevant nuances across different milk value chains in Bhutan [52–54].

As bTB has been confirmed in Bhutanese herds [12], the results of this study support a series of short- and mid-term recommendations. The severity of knowledge gaps and risky practices identified in our study necessitate immediate action to prevent potential zoonotic transmission and protect both animal and human health. First, a national cross-sectional study to determine the prevalence of bTB in herds, accompanied by a KAP survey, should be prioritised to comprehend the bTB landscape of Bhutan and the sociocultural, political, and economic factors that shape the prevalence and distribution of animal and human bTB across the country. The low awareness of bTB among farmers and consumers [50] found in our studies could favour the spread of bTB nationally, increasing the risk of zoonotic infection and reducing animal well-being and farmers’ livelihoods. A national prevalence and KAP study will identify bTB hotspots that can be intervened with targeted campaigns that account for regional variations and associated distinct farmer typologies identified. Second, a pilot herd surveillance program in high-risk dzongkhags should be established, underpinned by local veterinary staff trained in bTB testing, reporting, and monitoring. This step is crucial as animal culling for disease control is generally avoided in Bhutan [55], and alternative mitigation strategies beyond testing and slaughter are required [1]. In the mid-term, Bhutan should move towards a national One Health bTB surveillance program that integrates into existing agricultural extension programs and leverages the capacity of the existing national tuberculosis program, focused on human tuberculosis [56]. Besides bTB surveillance, pasteurisation should be prioritised nationwide to reduce the risk of bTB and other milk-borne diseases.

The geographic clustering of knowledge, attitudes, and practices we identified suggests that a one-size-fits-all approach to bTB control will likely be ineffective in Bhutan. Instead, strategies should be customised to local conditions, focusing on resource limitations and traditions in remote areas while taking advantage of higher baseline knowledge and risk perception near urban centres. The paradoxical relationship between risk perception and behaviour we observed underscores the importance of interventions that tackle structural obstacles to safe practices, beyond simply increasing community knowledge.

## Supporting information

S1 Text Appendix A: Knowledge, Attitudes and Practices Survey. Appendix B: Frequencies of knowledge, attitudes, and practices stratified by Dzongkhags. Appendix C: Dimensional reduction and data clustering.
(DOC)

S1 Data Farmers data. (CSV)
