# Supplementary material for: Dairy farmers have minimal knowledge of bovine tuberculosis: A cross-sectional study in Bhutan
Source: PLoS Negl Trop Dis. 2025 Dec 22;19(12):e0013817. doi: 10.1371/journal.pntd.0013817 (PMC12721510; doi:10.1371/journal.pntd.0013817)
Supplement: S1 Text — Appendix B: Frequencies of knowledge, attitudes, and practices stratified by Dzongkhags. Appendix C: Dimensional reduction and data clustering. (DOC) [file pntd.0013817.s001.doc]

**Dairy Farmers Have Minimal Knowledge of Bovine Tuberculosis: A Cross-Sectional Study in Bhutan**

# Juan-Pablo Villanueva-Cabezas^1,2^, Sithar Dorjee^3^, Justin McKinley^2^, Rinzin Pem^4^, Sangay Rinchen^4^

**Supplementary materials**

| **Appendix A. Knowledge, Attitudes and Practices Survey**  **KAP Farmer** | | | *Page 1* |  | |
| --- | --- | --- | --- | --- | --- |
|  |  | |
| འོག་ལུ་ཡོད་པའི་བརྟག་ཞིབ་ཀྱི་དྲི་བ་ཚུ་མཇུག་བསྡུ་གནང་ཞུ། བཀྲིན་ཆེ། | | |  |  | |
| བརྟག་ཞིབ་ཀྱི་དུས་ཡུན་ཆུ་ཚོད་ | | |  |  | |
| ཁྱོད་ཀྱི་མིང་ག་ཅི་སྨོ? | | |  |  | |
|  | | | ཕོ། |  | |
| ཕོ་མོའི་མཚན་རྟགས | | | མོ། |  | |
|  | | | ཕོ་མོའི་མཚན་རྟགས་སླབ་ནི་ལས་འཛེམ་ནི། |  | |
|  | | |  |  | |
| སྐྱེས་ལོ | | |  |  | |
| ལོ་སླབ་ནི་ལས་འཛེམ་ནི། | | |  | |
|  |  | |
| ཁྱོད་རྒེད་འོག་ག་སྟེ་ལུ་སྡོཔ་སྨོ་ | | |  |  | |
| རྒེད་འོག་སླབ་ནི་ལས་འཛེམ་ནི། | | |  | |
|  |  | |
| སློབ་སྦྱོང་ཤེས་ཚད། | | | སློབ་གྭ་ཆུང་བ སློབ་རིམ་༡ལས༦ པ་ཚུན། |  | |
|  | | | སློབ་གྭ་བར་མ་ སློབ་རིམ་༦-༨ཚུན་ |  | |
|  | | | (འབྲིང་རིམ་བར་མ་ སློབ་རིམ་༨-༡༠ཚུན་ |  | |
|  | | | སློབ་གྭ་ཆེ་བ་ སློབ་རིམ་༡༠-༡༢ ཚུན་ |  | |
|  | | | མཐོ་རིམ་ཤེས་ཚད་ |  | |
| སྟེང་འཇག་ཁྱོད་ཀྱི་ཁྱིམ་ནང་ མི་ག་དེ་གཅི་ ཡོད (ཨ་རྟག་ཁྱིམ་ནང་ཉལ་མི) | | |  |  | |
|  | | |  |  | |
|  | | | ལོ་༠ དང་༥འི་བར་ |  | |
|  | | | ལོ་༥་དང་༡༠་གྱི་བར |  | |
|  | | | ལོ༡་༠ དང་༡༥འིིི་བར་ |  | |
| ཚོང་ལས་ཀྱི་དོན་ལུ་ཁྱོད་སྒོ་ནོར་སེམས་ཅན་གསོ་སྟེ་ལོ་ངོ་ག་དེ་ཅིག་སོང་ཡི? | | | ལོ་༡༥ དང་༢༠འི་བར་ |  | |
|  | | | ལོ་༢༠ ལྷག་ཙམ |  | |
| ཁྱོད་ལུ་སྒོ་ནོར་སེམས་ཅན་ཡོད་ག­? | | | ནོར |  | |
|  | | | ར |  | |
|  | | | ཕགཔ |  | |
|  | | | གཡག |  | |
|  | | | མ་ཧེ |  | |
|  | | | ལུག |  | |
|  | | | བོང་ཀུ |  | |
|  | | | བྱ |  | |
| ཁྱོད་ལུ་ ནོར་དང་གཡག་ཡོད་པ་ཅིན་ ཡོངས་བསྡོམས་ག་དེམ་ཅིག་ཡོད་  མེད་པ་ཅིན་ ༠ བཙུགས | | |  |  | |
| ཁྱོད་ལུ་བ་ནོར་དང་གཡག་ཡོད་ག་ ཁ་གྱངས་བཙུགས་གནང་ མེད་པ་ཅིན་གླེན་སྒོར་བཙུགས | | |  |  | |
| **བམ་ཚན་འདི་གི་ང་བཅས་མི་ལུ་བད་ཀན་གློ་གཙོང་གི་སྐོར་ལས་ཡོན་ཏན་གྱི་དྲི་བ་ཨིན།** | | | |  | |
| **K1** | ཁྱོདཀྱི་**བད་ཀན་ནད་གཞིའི་སྐོར་ལས་གོ་ཡི་ག­་ ཤེས་ཅིག་ག**? | གོ་ཡི | |  | |
|  |  | མ་གོ་ | |  | |
|  |  | ང་མི་ཤེས་ | |  | |
| **K2** | ད་ལྟོ་གི་དྲི་བ་དྲི་ལན་མ་འབད་བའི་ཧེན་མ་ མི་ཚུ་ལུ་བད་ཀན་གྱི་ནད་གཞི་ཐོབ་ཐོབ་འོང་ཟེར་མནོ་ཡི་ག་ | ཤེས་ མི་ཤེས་ ང་མ་ཤེས་ | |  | ཤེས་ མི་ཤེས་ ང་མ་ཤེས་ |

|  |  | *Page 2* | | | | | | | |
| --- | --- | --- | --- | --- | --- | --- | --- | --- | --- |
| K3 | འོག་ལུ་ཡོད་པའི་དྲི་བ་ཚུ་ ལྟ་ལྟོགས་འབད་འདི་ འོས་འབབ་ཡོད་མི་ཚུ་རྟགས་བཀལ་ ང་བཅས་མི་ལུ་བད་ཀན་གྱི་ནད་གཞི་འབུ་ནིའི་ལམ་ག་ཅིག་ར་ཡོད་ག་སྨོ? | ཐད་དཀར་དུ་བད་ཀན་གྱི་ནད་གཞི་ཡོད་པའི་མི་དང་ཅིག་ཁར་འབྲེལ་བ་འཐབ་ནི | | | | | | | |
|  |  | ཐད་དཀར་དུ་བད་ཀན་གྱི་ནད་གཞི་ཡོད་པའི་ནོར་དང་གཡག་དང་ཅིག་ཁར་འབྲེལ་བ་འཐབ་ནི་ | | | | | | | |
|  |  | ཨོམ་མ་སྐོལཝ་འཐུང་ནི་ | | | | | | | |
|  |  | ཨོམ་མ་སྐོལཝ་གི་ཞོའཐུང་ནི་ | | | | | | | |
|  |  | ཨོམ་མ་སྐོལཝ་གི་དར་ཚི་ཟ་ནི་ | | | | | | | |
|  |  | ཤ་མ་བཙོཝ་ཟ་ནི་ | | | | | | | |
|  |  | རྨ་གཙོག་པ་ལས་ | | | | | | | |
|  |  | བུ་ཚང་ལག་བཟུང་ | | | | | | | |
|  |  | ང་མི་ཤེས་ | | | | | | | |
|  |  | ལྟ་ཀོ་སྦོ་ནི་ | | | | | | | |
|  |  | གློ་འབར་ནི་ | | | | | | | |
| K4 | འོག་ལུ་ཡོད་པའི་དྲི་བ་ཚུ་ ལྟ་ལྟོགས་འབད་འདི་ འོས་འབབ་ཡོད་མི་ཚུ་རྟགས་བཀལ་ ང་བཅས་མི་ལུ་བད་ཀན་གྱི་ནད་གཞི་ཐོབ་མི་ཚུ་གིས་རྟགས་ཚན་ག་ཅིག་ར་བཏོནམ་སྨོ? | གློ་འབར་འདི་བདུན་ཕྲག་གསུམ་ལས་ལྷག་སྟེ་སྡོད་ནི་ | | | | | | | |
|  |  | བྱག་ཁོག་ན་ཟུག་རྐྱབ་ནི་ | | | | | | | |
|  |  | དྲོད་འབར་རྐྱབ་ནི་ | | | | | | | |
|  |  | ཟ་ཁམས་མེདཔ་འགྱོ་ནི་ | | | | | | | |
|  |  | ལྗིད་ཚད་མར་འབབ་ | | | | | | | |
|  |  | ང་མི་ཤེས་ | | | | | | | |
|  | | | | | | | | | |
| **ཁྱོད་རའི་ཡོན་ཏན་གྱི་གཞི་དང་འཁྲིཝ་དང་ འོག་ལུ་ཡོད་མི་ཚུ་ཁྱོད་ཀྱི་ག་དེ་འབད་ཆ་བཞག་ནི་སྨོ** | | མ་པ་ལས་མི་བདེན | | མི་བདེན་ | | བདེན་ | | ངེས་བདེན་ཨིན | |
| K5 | མི་བད་ཀན་གྱི་ནད་གཞི་ཐོབ་མི་ཚུ་གཟུགས་ཁམས་སྒྲིང་སྒིང་འབད་མཐོང་འོང་ག་ |  | |  | |  | |  | |
| K6 | བད་ཀན་གྱི་ནད་གཞི་ཐོབ་མི་ཚུ་སྨན་བཅོས་འབད་ཞིནམ་ལས་དྲག་ནིའི་གོ་སྐབས་ཡོད་ |  | |  | |  | |  | |
| K7 | འབྲུག་ལུ་ང་བཅས་རང་མི་སེར་ཚུ་ལུ་བད་ཀན་གྱི་ནད་གཞི་བརྟག་དཔྱད་འབད་ནིའི་གོ་སྐབས་ཡོད་ |  | |  | |  | |  | |
| K8 | འབྲུག་ལུ་ང་བཅས་རང་མི་སེར་ཚུ་ལུ་བད་ཀན་གྱི་ནད་གཞི་སྨན་བཅོས་འབད་ནིའི་གོ་སྐབས་ཡོད་ |  | |  | |  | |  | |
| K9 | འབྲུག་ལུ་ང་བཅས་རང་མི་སེར་ཚུ་ལུ་བད་ཀན་གྱི་ནད་གཞིའི་སྨན་ཁབ་བཙུགས་ནིའི་གོ་སྐབས་ཡོད་ |  | |  | |  | |  | |
| K10 | ང་བཅས་རང་མི་ཚུ་ལུ་བད་ཀན་གྱི་ནད་གཞི་གི་སྨན་བཅོས་དྲག་ཤོས་ར་ག་ཅིག་སྨོ? | དེང་སང་དང་ཕྱིར་བའི་སྨན་ | | | | | | | |
|  |  | སྔར་སྲོལ་དང་ནང་སྨན་ | | | | | | | |
|  |  | གཉིས་ཆ་རང་ | | | | | | | |
|  |  | སྨན་བཅོས་འབད་མི་ཚུགས | | | | | | | |
|  |  | ང་མི་ཤེས་ | | | | | | | |
| K11 | འོག་ལུ་ཡོད་པའི་དྲི་བ་ཚུ་ ལྟ་ལྟོགས་འབད་འདི་ འོས་འབབ་ཡོད་མི་ཚུ་རྟགས་བཀལ་ ང་བཅས་རང་བད་ཀན་གྱི་ནད་གཞི་བཀག་ཐབས་ཀྱི་དོན་ལུ་དང་ལེན་ག་ཅིག་རང་འབད་དགོཔ་འདུག? | བད་ཀན་གྱི་ནད་གཞི་ཐོབ་མི་ཚུ་དང་འབྲེལ་བ་འཐབ་ནི་ལསའཛེམ་ | | | | | | | |
|  |  | འཕྲལ་འཕྲལ་རང་ལགཔ་འཁྱུ་དགོ | | | | | | | |
|  |  | ཨོམ་བསྐོལ་ཞིནམ་ལས་བཟོ་བསྐྲུན་འབད་ཡོད་པའི་ཞུ་འཐུང་ནི་ | | | | | | | |
|  |  | ནད་གཞི་ཐོབ་ཡོད་མིནོར་ཚུ་དང་འབྲེལ་འཐབ་ནི་ལས་འཛེམ་ ༼ལགཔ་རེག་ནི་དང་ཨོམ་འཐུང་ནི༽ | | | | | | | |
|  |  | ཤ་ལེགས་ཤོམ་འབད་བཙོ་ཟ་ནི་ | | | | | | | |
|  |  | ག་ཅིག་ཡང་མེན་ | | | | | | | |
| K12 | འོག་ལུ་ཡོད་པའི་དྲི་བ་ཚུ་ ལྟ་ལྟོགས་འབད་འདི་ འོས་འབབ་ཡོད་མི་ཚུ་རྟགས་བཀལ་ ང་བཅས་མི་ཚུ་ལུ་བད་ཀན་གྱི་ནད་གཞི་ འབུ་ནི་ལས་བཀག་ཐབས་ཀྱི་ཐབས་ཤེས་ག་ཅིག་རང་འབད་དགོཔ་འདུག? | གློ་འཁོག་བའི་སྐབས་ཁ་དང་ཧ་པ་སྤུབ་ནི་ | | | | | | | |
|  |  | སྨན་ཁབ་བཙུགས་ནི | | | | | | | |
|  |  | འབུ་ནི་ལས་བཀག་ཐབས་ཀྱི་ཐབས་ཤེས་ག་ཅིག་རང་འབད་དགོཔ་འདུག? | | | | | | | |
|  |  | ནདཔ་ཟུར་གནས་བཞག་ནི | | | | | | | |
|  |  | ཐབ་ཆས་བླ་སྲེས་ནི་ལས་འཛེམ་ | | | | | | | |
|  |  | སྔ་གོང་དུས་ཚོད་ཁར་ནད་སྨན་བཅོས་འབད་ནི་ | | | | | | | |
|  |  | ཚད་བཟུང་བཞེས་སྒོ་ཟ་ནི་ | | | | | | | |
| **བམ་ཚན་འདི་ནང་ ཡོན་ཏན་གྱི་དྲི་བ་འདི་ ནོར་དང་གཡག་ནང་ལུ་བད་ཀན་གྱི་སྐོར།** | | | | | | | | | |
| K13 | ད་ལྟོ་གི་དྲི་བ་དྲི་ལན་མ་འབད་བའི་ཧེན་མ་མི་ཚུ་ལུ་ནོར་དང་གཡག་ཚུ་ལས་བད་ཀན་གྱི་ནད་གཞི་འབུ་འོང་ཟེར་མནོ་ཡི? | | མནོ་ཡི་ | | | | | | |
|  |  |  | མ་མནོ་ | | | | | | |
|  |  |  | ང་མ་བཤེས་ | | | | | | |
| K14 | འོག་ལུ་ཡོད་པའི་དྲི་བ་ཚུ་ ལྟ་ལྟོགས་འབད་འདི་ འོས་འབབ་ཡོད་མི་ཚུ་རྟགས་བཀལ་ ནོར་དང་གཡག་ཚུ་ཡང་བད་ཀན་གྱི་ནད་གཞི་ཐོབ་འོང་ཟེར་ཧ་གོ་ཡི་ག? | | ནད་འབུབ་དང་ཕྲ་འབུབ་ཚུ་ཕོཝ་ནང་ལུ་བཞུ་ནི་དང་ཐིམ་ནི་ | | | | | | |
|  |  |  | ནད་གཞི་ཡོད་མི་སེམས་ཅན་དང་ཐད་དཀར་དུ་འབྲེལ་བ་འཐབ་ནི་ | | | | | | |
|  |  |  | གོང་གི་འདི་ཚུ་ག་ཡང་མེན་ | | | | | | |
|  |  |  | ང་མི་ཤེས་ | | | | | | |
| K15 | འོག་ལུ་ཡོད་པའི་དྲི་བ་ཚུ་ ལྟ་ལྟོགས་འབད་འདི་ འོས་འབབ་ཡོད་མི་ཚུ་རྟགས་བཀལ་ ནོར་དང་གཡག་ཚུ་ལུ་བད་ཀན་གྱི་ནད་གཞི་ཐོབ་དང་རྟགས་ཚན་ག་ཅིག་རབཏོནམ་སྨོ? | | དྲོད་འབར་ | | | | | | |
|  |  |  | གཟུགས་ཀྱི་ལྗིད་ཚད་མར་འབབ་འགྱོ་ནི་ | | | | | | |
|  |  |  | གཟུགས་ཀྱི་སྤུ་རྩུབ་དྲགས་འགྱོ་ནི་ | | | | | | |
|  |  |  | བདུན་ཕྲག་དག་པ་ཅིག་ ཨུ་ཚུགས་ཅན་གྱི་གློ་འཁོག་ནི་ | | | | | | |
|  |  |  | འབུངས་འགམ་ནི་ | | | | | | |
|  |  |  | ལྷ་པ་ནང་ལས་ལྷཔ་འཐོན་ནི་ | | | | | | |
|  |  |  | ལྟག་ཀོ་སྦོ་ནི་ | | | | | | |
|  |  |  | ཡང་ཅིན་བརྟག་ཚན་ག་ཅིག་ཡང་མི་འོང་ | | | | | | |
|  |  |  | གོང་གི་འདི་ག་ཡང་མེན་ | | | | | | |
|  |  |  | ང་མི་ཤེས་ | | | | | | |
| **ཁྱོད་རའི་ཡོན་ཏན་གྱི་གཞི་དང་འཁྲིལ་ འོང་ལུ་བཀོད་མི་ཚུ་ལུ་ངེས་བྱུང་ག་དེ་འབད་རང་ཡོད**? | | | | | | | | | |
|  |  | | མ་པ་ལས་མི་བདེན་ | | མི་བདེན་ | | བདེན་ | | ངེས་བདེན་ཡིན་ |
| K16 | ནོར་དང་གཡག་བད་ཀན་གྱིར་ནད་གཞི་ཐོབ་ཡོད་མི་ཚུ་གཟུགས་ཁམས་སྒྲིང་སྒྲིང་མཐོང་འོང་ | |  | |  | |  | |  |
| K17 | ནོར་དང་གཡག་བད་ཀན་གྱི་ནད་གཞི་ཐོབ་ཡོད་མི་ཚུ་ལོ་ལེ་ཤཱ་སྡོད་འོང་༼ཚེ་རིངམ་ཐུབ༽ | |  | |  | |  | |  |
| K18 | བད་ཀན་གྱི་ནད་གཞི་ཐོབ་མི་ནོར་དང་གཡག་ཚུ་སྨན་བཅོས་མ་འབད་བར་རང་བཞིན་གྱི་དྲག་འོང་ | |  | |  | |  | |  |
| K19 | བ་ནོར་གྱི་བད་ཀན་ནད་གཞི་ཐོབ་མི་ནོར་དང་གཡག་ཚུ་ཤི་འོང་ | |  | |  | |  | |  |
| K20 | འོག་ལུ་ཡོད་པའི་དྲི་བ་ཚུ་ ལྟ་ལྟོགས་འབད་འདི་ འོས་འབབ་ཡོད་མི་ཚུ་རྟགས་བཀལ་ ནོར་དང་གཡག་ཚུ་ལུ་བད་ཀན་གྱི་ནད་གཞི་དར་ཁྱབ་འགྱོ་ནི་ལས་ག་དེ་འབད་བཀག་ཆགས་འབད་ནི? | | སྨན་ཁབ་བཙུགས་ནི་ | | | | | | |
|  |  |  | སྤགས་ཀོ་གུར་བད་ཀན་གྱི་ནད་གཞི་བརྟག་དཔྱད་འབད་ནི་ | | | | | | |
|  |  |  | བད་ཀན་གྱི་ནད་གཞི་ཐོབ་མི་སེམས་ཅན་ཚུ་ཟུར་གནས་བཞག་ནི་ | | | | | | |
|  |  |  | ག་ཅིག་ཡང་མེན་ | | | | | | |
|  |  |  | ང་མི་ཤེས་ | | | | | | |
| K21 | ཁྱོད་རའི་བ་ནོར་གསོ་སྐྱོང་ཁང་ནང་ལུ་ ནོར་དང་གཡག་ཕར་ཚུར་འགྲོ་འགྲུལ་དང་ གསརཔ་མཁོ་སྒྲུབ་འབད་ནིཚུ་གིས་ བད་ཀན་གྱི་ནད་གཞི་དར་ཁྱབ་འགྱོ་འོང་ག? | | འགྱོ་འོང་ | | | | | | |
|  |  |  | མི་འགྱོ | | | | | | |
|  |  |  | ང་མི་ཤེས | | | | | | |
| **དྲི་བ་འདི་སྤྱོད་ལམ་གྱི་སྐོར་ཨིན་ མི་ལུ་བད་ཀན་གྱི་ནད་གཞིའི་སྐོར་ལས་དམིགས་བསལ་དུ་ཨིན།**  **ཁྱོད་རའི་ཉམས་མྱོང་དང་ངེས་བྱུང་ལས་རྟེན་ག་དེམ་ཅིག་ཆ་བཞག་འོང་** | | | | | | | | | |
|  |  | | མ་པ་ལས་མི་བདེན་ | | མི་བདེན་ | | བེདན་ | | ངེས་བདེན |
| A22 | འབྲུག་ལུ་མི་སེར་ཚུ་བད་ཀན་གྱི་སྨན་ཁབ་བཙུགས་ནི་འདི་གིས་ང་རང་དང་མི་སྡེ་ནང་ལུ་བད་ཀན་གྱི་ནད་གཞི་བཀག་འཛིན་འབད་དོ་ | |  | |  | |  | |  |
| A23 | མི་ག་ར་འབད་རུང་བད་ཀན་གྱི་ནད་གཞི་གི་བརྟག་ཚན་བཏོན་མ་ཅིན་ འདི་འཕྲོད་ལས་སྒོ་ནོར་གསོ་བའི་བསླབ་བྱ་ལེན་དགོ་ | |  | |  | |  | |  |
| A24 | བད་ཀན་གྱི་ནད་གཞི་འདི་འབྲུག་ལུ་ མི་མང་ལུ་ཁག་ཆེ་བའི་ནད་གཞི་ཅིག་ཨིན་ | |  | |  | |  | |  |
| A25 | བད་ཀན་གྱི་ནད་གཞི་འདི་མི་མཉམ་ཆུང་ལུ་རྐྱངམ་ཅིག་འབུར་འོང་ | |  | |  | |  | |  |
| A26 | བད་ཀན་གྱི་ནད་གཞི་ཐོབ་མི་ཚུ་ལུ་ ཁྱོད་ཀྱི་མནོ་བསམ་མང་ཆེ་བ་ག་དེ་འབད་རང་བཏང་དོ? | | སྙིང་རྗེ་ཆེ་བ | | | | | | |
|  |  |  | སྙིང་རྗེ་ཡོད་དོ་བཟུམ | | | | | | |
|  |  |  | མནོ་བསམ་ག་ཅིག་ཡང་མེད་ | | | | | | |
|  |  |  | འཇིགས་སྣང་ཅན་མས | | | | | | |
| A27 | བད་ཀན་གྱི་ནད་གཞི་ཐོབ་མི་ལུ་ཉམས་མྱོང་འ་ནི་བཟུམ་ཐོབ་འོང་ | | མ་དུངལ་མ་ལངས་པའི་དཀའ་ཐུབ | | | | | | |
|  |  |  | གཟུགས་ཁམས་ཀྱི་དཀའ་ཐུབ | | | | | | |
|  |  |  | མི་སྡེ་ནང་ལུ་དཀའ་ཐུབ | | | | | | |
|  |  |  | གོང་འཁོད་ཡོད་མི་ཚུ་ཅིག་ཡང་མེན་ | | | | | | |
|  |  |  | གཞན | | | | | | |
| A28 | གོང་གི་དྲི་བ་ནང་གཞན་ཟེར་འཁོད་ཡོད་པ་ཅིན་ རྒྱབ་ཁུངས་བཀལ་གནང་ | |  | | | | | | |
| **དྲི་བ་འདི་སྤྱོད་ལམ་གྱི་སྐོར་ཨིན་ ནོར་དང་གཡག་ལུ་བད་ཀན་གྱི་ནད་གཞིའི་སྐོར་ལས་དམིགས་བསལ་དུ་ཨིན།** | | | | | | | | | |
| A29 | མིའི་སྡཻ་ཚན་ག་ལུ་ བ་ནོར་ལུ་ཡོད་པའི་བད་ཀན་གྱི་ནད་གཞི་ཐོབ་ནིའི་ཉན་ཁག་ཡོད? | | མི་སེར་བ་ནོར་འཚོ་སྐྱོང་འཐབ་མི་ | | | | | | |
|  |  |  | ནོར་གྱི་ཐོན་སྐྱེད་ཚོང་འབྲེལ་འཐབ་མི | | | | | | |
|  |  |  | ནོར་གྱི་ཐོན་སྐྱེད་ཟ་མི | | | | | | |
|  |  |  | གོང་གི་ག་ར་ཨིན | | | | | | |
|  |  |  | ག་ཡང་མེན་ | | | | | | |
|  |  |  | ང་མི་ཤེས | | | | | | |
| **ཁྱོད་རའི་སྤྱོད་ལམ་དང་རྟོགས་པ་དང་འཁྲིལ་ ཁྱོད་ཀྱི་ག་དེ་འབད་ངོས་ལེན་འབད་ནི་སྨོ་** | | | | | | | | | |
|  |  | | མ་པ་ལས་མི་བདེན | | མི་བེདན | | བེདེན | | ངེས་བདེན |
| A30 | ནོར་གྱི་བད་ཀན་ནད་གཞི་འབུར་ནི་འདི་ ནོར་དང་གཡག་དང་གཅིག་ཁར་ཐད་དཀར་དུ་འབྲེལ་བ་འཐབ་ནི་དང་རེག་ནི་ལས་ཉན་ཁག་མང་སུ་ཡོད་ | |  | |  | |  | |  |
| A31 | བད་ཀན་གྱི་ནད་གཞི་ཐོབ་ཡོད་པའི་སེམས་ཅན་ལས་ཨོམ་མ་བསྐོལཝ་དང་མར་ དར་ཚི་ཞོ་ ཟ་བ་ཅིན་ བད་ཀན་གྱི་ནད་གཞི་ཐོབ | |  | |  | |  | |  |
| A32 | ནོར་ཤ་མ་བཙོཝ་ཟ་ནི་ཚུ་ལས་ མི་ལུ་བ་ནོར་གྱི་བད་ཀན་ཐོབ་ནིའི་ཉན་ཁག་ཡོད | |  | |  | |  | |  |
| A33 | ནོར་དང་གཡག་ནདཔ་ཚུ་སེམས་ཅན་གཞན་ལས་ཟུར་གནས་སོར་སོ་ནང་བཞག་དགོ | |  | |  | |  | |  |
| A34 | ངིའི་ནོར་དང་གཡག་ཚུ་བ་ནོར་གྱི་བད་ཀན་ནད་གཞི་ཐོབ་སྟེ་ན་ཟུགས་ཕོག་འོང་ | |  | |  | |  | |  |
| A35 | འོག་གི་དོན་ཚིག་ཚུ་དབྱེ་དཔྱད་འབད་འདི་རྟགས་བཀལ་ གལ་སྲིད་ནོར་དང་གཡག་ཚུ་ནད་གཞི་གཞན་ལས་རྟེན་ཤི་རྐྱེན་འབྱུང་ནང་༼རང་བཞིན་དང་ལོ་ན་རྒས་ གཞན་ཡང་རྐྱེན་ལས་རྟེན་ཤི་ན༽ | | མི་ཚུ་གིས་ཟ་འོང་ | | | | | | |
|  |  | | མ་ཟ་བར་ ས་དོང་ནང་ལུ་བཙུགས་བཞག་འོང་ | | | | | | |
|  |  | | མ་ཟ་བར་ དེ་སྦེ་རང་རུལ་བཅུག་ནི་ | | | | | | |
|  |  | | ང་མི་ཤེས | | | | | | |
| A36 | འོག་གི་དོན་ཚིག་ཚུ་དབྱེ་དཔྱད་འབད་འདི་རྟགས་བཀལ་ ཁྱོད་ཀྱི་ནོར་དང་གཡག་ཚུ་བད་ཀན་གྱི་ནད་གཞི་ཡོདཔ་འབད་ངོས་འཛིན་འབད་ཡོད་པ་ཅིན་ ཁྱོད་ཀྱི་ག་དེ་འབད་སྦེ་འོང་? | | སེམས་ཅན་འདི་བརྩོང་འོང་ | | | | | | |
|  |  | | ང་རང་གི་སྨན་བཅོས་འབད་འོང་ | | | | | | |
|  |  | | སྒོ་ནོར་གྱི་རྒྱབ་སྐྱོར་འཚོལ་ནི | | | | | | |
|  |  | | གཞན་ | | | | | | |
| A37 | གོང་གི་དྲི་བ་ནང་ལུ་ཁྱོད་ཀྱི་གཞན་ཟེར་བཀོད་པ་ཅིན་ འབྲེལ་བཤད་རྐྱབ་གནང་ | |  | | | | | | |
| **དྲི་བ་འདི་ ང་བཅས་མི་ནང་ལུ་བད་ཀན་གྱི་ནད་གཞིའི་སྐོར་** | | | | | | | | | |
| P38 | སྤྱིར་བཏང་ཁྱོད་ཀྱི་ཨོམ་ག་དེ་འབད་འཐུངམ་སྨོ? | | མ་བསྐོལ་བ (ཨོམ་དང་ཞོ) | | | | | | |
|  |  | | བསྐོལ་ཞིནམ་ལས | | | | | | |
|  |  | | འཕྲུལ་ཆས་ཐོག་ཨོམ་བསྐོལ་ཞིནམ་ལས་འཐུང་ནི་ | | | | | | |
|  |  | | ཨོ་ཕྱེ་ | | | | | | |
|  |  | | ང་ཨོམ་འཐུང་ནི་མེད་ | | | | | | |
| P39 | ཁྱོད་ཀྱི་ཨོམ་བསྐོལ་ཞིནམ་ལས་འཐུང་མི་ཟེར་གདམ་ཁ་རྐྱབ་པ་ཅིན་ ག་ཅིག་འབད་བསྐོལ་ཞིནམ་ལས་འཐུང་དགོཔ་སྨོ | | ནད་གཞི་བཏོན་བཏང་ནི | | | | | | |
|  |  | | བྲོཝ་ཐེབས་འོང་ནི་ | | | | | | |
|  |  | | ངིའི་བཟའ་ཚང་ནང་ཨ་བརྟག་རང་ཡོད་ | | | | | | |
|  |  | | ང་ཨོམ་ཚ་ཏོམ་དགའ་བས་ | | | | | | |
| P40 | ཁྱོད་ཀྱི་ཨོམ་ཚར་ག་དེ་རེ་འཐུང་ནི་ཡོད? | | བདུན་ཕྲག་རེ་ཚར་རེ་ | | | | | | |
|  |  | | བདུན་ཕྲག་གཅིག་ནང་ཚར་ལེ་ཤཱ་ | | | | | | |
|  |  | | ཉིན་ལྟར་ཞིན་དུ་ | | | | | | |
|  |  | | བརྒྱ་ལམ་རེ་ (དགའ་སྟོན་དང་འཁྲིལ) | | | | | | |
| P41 | ཁྱོད་ཀྱི་བ་ནོར་གྱི་ཐོན་སྐྱེད་ནམ་རང་ཟཝ་སྨོ(དཔེར་ན་ དར་ཚི་ མར་ ཞོ | | བདུན་ཕྲག་གཅིག་ནང་ཚར་ལེ་ཤཱ་ | | | | | | |
|  |  | | ཉིན་ལྟར་ཞིན་དུ་ | | | | | | |
|  |  | | བརྒྱ་ལམ་རེ་ | | | | | | |
|  |  | | ཟ་ནི་ར་མེད་ | | | | | | |
| P42 | འོག་གི་དྲི་བ་ཚུ་ དབྱེ་དཔྱད་འབད་དེ་བརྟགས་བཀལ་ | | ཁྱིམ་ནང་བཟོ་བསྐྲུན་ | | | | | | |
|  | ཁྱོད་ཀྱི་སྒོ་ནོར་གྱི་ཐོན་སྐྱེད་ཚུ་འབྱུང་ཁུངས་ག་སྟེ་ལས་སྨོ་? (དཔེར་ན་ དར་ཚི་ མར་ ཞོ) | | ཁྲོམ་ཁར་ལས་ | | | | | | |
|  |  | | བཟའ་ཚང་/ཆ་རོགས་ལས་ | | | | | | |
|  |  | | གཡུས་ཁ་ལས་ | | | | | | |
|  |  | | གཞན་ | | | | | | |
| P43 | གོང་གི་དྲི་བ་ནང་ ཁྱོད་ཀྱི་གཞན་ཁ་ལས་ཟེར་གདམ་འཐུ་འབད་བ་ཅིན་ རྒྱབ་ཁུངས་བཀལ་གནང་ | |  | | | | | | |
| P44 | སྤྱིར་བཏང་ཁྱོད་ཀྱི་ནོར་ཤ་ག་དེ་འབད་ཟཝ་སྨོ? | | མ་བཙོ་བ | | | | | | |
|  |  | | བཙོ་ཞིནམ་ལས | | | | | | |
|  |  | | ང་ནོར་ཤ་ར་མི་ཟ་ | | | | | | |
| P45 | ཁྱོད་ལུ་ནོར་ཤ་གི་འབྱུང་ཁུངས་ག་སྟེ་ལས་སྨོ? | | ཁྲོམ་ཁར | | | | | | |
|  |  | | བཟའ་ཚང་/ཆ་རོགས་ལས | | | | | | |
|  |  | | གཡུས་ཁ་ལས (ཁྱིམ་ཚང་) | | | | | | |
|  |  | | གཞན | | | | | | |
| P46 | གོང་གི་དྲི་བ་ནང་ ཁྱོད་ཀྱི་གཞན་ཁ་ལས་ཟེར་གདམ་འཐུ་འབད་བ་ཅིན་ རྒྱབ་ཁུངས་བཀལ་གནང་ | |  | | | | | | |
| P47 | ཁྱོད་ཀྱི་འཐུང་ཆུའི་རྐ་འདི་ ནོར་དང་གཡག་ཚུ་ལུ་བྱིན་མི་ཆུའི་རྐ་འདི་རང་ཨིན་ན?  དཔེར་ན་ ཁྱོད་ཀྱི་འཐུང་ཆུ་འདི་ ནོར་དང་གཡག་ཚུ་འཐུང་མིའི་ཆུའི་རྐ་འདི་ནང་ལས་དབོགས་སྦགས་འོངམ་ཨིན་ན? | | ཨིན | | | | | | |
|  |  | | མེན | | | | | | |
|  |  | | ང་མི་ཤེས | | | | | | |
| P48 | ཁྱོད་ཀྱི་གཟུགས་ཁར་ བད་ཀན་གྱི་ནད་གཞི་ཐོབ་ཆེཝ་བཟུམ་ཅིག་འབད་བ་ཅིན་ ཁྱོད་ཀྱི་ག་ཅིག་རང་འབད་འོང་? | | སྨན་ཁང་ནང་འགྱོ་ནི | | | | | | |
|  |  | | གཡུས་ཁའི་སྨན་ཁང་ནང་འགྱོ་ནི་ | | | | | | |
|  |  | | སྔར་སྲོལ་གྱི་སྨན་བཅོས་འབད་མི་ཚུ་འབད་སར་འགྱོ་ནི་ | | | | | | |
|  |  | | ག་ཅིག་ཡང་མི་འབད་ | | | | | | |
|  |  | | གཞན་ | | | | | | |
| P49 | གོང་གི་དྲི་བ་ནང་ ཁྱོད་ཀྱི་གཞན་ཁ་ལས་ཟེར་གདམ་འཐུ་འབད་བ་ཅིན་ རྒྱབ་ཁུངས་བཀལ་གནང་ | |  | | | | | | |
| P50 | ཁྱོད་ཀྱི་ནོར་དང་གཡག་ཨོམ་ཞོ་མི་ཚུ་དང་ཐད་དཀར་དུ་འབྲེལ་བ་འཐབ་ནི་དང་ལགཔ་རེག་ཞིནམ་ལས་ ལགཔ་འཁྱུ་ནི་ཡོད་ག? | | འཁྱུ་ནི་ར་མེད | | | | | | |
|  |  | | བརྒྱ་ལམ་རེ་འཁྱུ་འོང་ | | | | | | |
|  |  | | འཕྲལ་འཕྲལ་འཁྱུ་འོང་ | | | | | | |
|  |  | | ཨ་བརྟག་རང་འཁྱུ་འོང་ | | | | | | |
|  |  | | ང་མི་ཤེས | | | | | | |
| P51 | ཁྱོད་རང་ལུ་བད་ཀན་གྱི་ནད་གཞི་བརྟག་དཔྱད་འབད་ཡི་ག? | | འབད་ཡི | | | | | | |
|  |  | | མ་འབད་ | | | | | | |
|  |  | | ང་མི་ཤེས་འབད་ | | | | | | |
| P52 | ཁྱོད་བད་ཀན་གྱི་ནད་གཞི་གི་དོན་ལས་སྨན་བཅོས་ཐོབ་ཅིག་ག? | | ཐོབ་ཅིག | | | | | | |
|  |  | | མ་ཐོབ་ | | | | | | |
|  |  | | ང་མི་ཤེས་འབད་ | | | | | | |
| P53 | ཁྱོད་རའི་བཟའ་ཚང་ནང་ལུ་བད་ཀན་གྱི་སྨན་ཁབ་ཀྱི་གནས་སྟངས་སྐོར་ | | ང་སྨན་ཁབ་བཙུགས་ཅིག | | | | | | |
|  | (bacille Calmette-Guérin vaccine)? | | ངིའི་ཨ་ལོ་ཚུ་ཡང་སྨན་ཁབ་བཙུགས་ཅིག་ | | | | | | |
|  |  | | ངིའི་བཟའ་ཚང་ག་ར་སྨན་ཁབ་བཙུགས་ཅིག་ | | | | | | |
|  |  | | ང་མི་ཤེས་འབད་ | | | | | | |
| P54 | ཁྱོད་སྨན་ཁང་ནང་ལུ་དུས་མཐུན་གྱི་བརྟག་དཔྱད་འབད་དོ་ག (ལོ་ཅིག་ལུ་ཚར་ཅིག)? | | འབད་དོ | | | | | | |
|  |  | | མ་འབད | | | | | | |
| **དྲི་བ་ཚུ་ཞིང་ལཱ་དང་བསྟུན་པའི་སྐོར་ ལན་ཨིན་དང་མེན་ཟེར་བཀོད་དགོ** | | | | | | | | | |
|  |  | | ཨིན | | | | མེན | | |
| P55 | ཁྱོད་ཞིང་ལཱ་འབད་བའི་སྐབས་སུ་ཉན་སྲུང་དང་ལྡན་པའི་གྱོན་ཆས་ལག་ལེན་འཐབ་ནི་ཡོད་ག (ཁའི་སྤུ་རས, ལག་ཤུབས, ཆུ་ལྷམ, གདོང་ཁེབས) | |  | | | |  | | |
| P56 | ཉིན་ལྟར་ཞིན་དུ་ཁྱོད་ཀྱི་བ་ནོར་གསོ་སྐྱོང་ཁང་ནང་ལུ་གཙང་ཏོག་ཏོ་འབད་བཞག་ནི་ཡོད་ག་ སྨན་རྫས་བླུགས་ཞིནམ་ལས་ནོར་གྱི་ཞག་ཉལ་ས་འཁྱུ་ནི་དང་ ནོར་འཁྱུ་འཁྱུ་ནི་ | |  | | | |  | | |
| P57 | ཁྱོད་རའི་བ་ནོར་གསོ་སྐྱོང་ཁང་ནང་ལཱ་འབད་ནི་འགོ་མ་བཙུགསཔ་པའི་ཧེན་མ་དང་་ ལཱ་འབད་ཚར་ཞིནམ་ལས་ལགཔ་འཁྱུ་ནི་ཡོད་ག? | |  | | | |  | | |
| P58 | ཁྱོད་ལུ་ཡོད་པའི་རྨ་ཚུ་ནོར་དང་གཡག་དང་འབྲེལ་བ་འཐབ་པའི་སྐབས་བདག་འཛིན་འབད་ནི་ཡོད་ག | |  | | | |  | | |
| P59 | ཁྱོད་ཀྱི་ནོར་དང་གཡག་ཚུ་ལུ་སྒོ་ནོར་གྱི་ཞབས་ཏོག་ག་གིས་བྱིནམ་སྨོ? | | སྒོ་ནོར་བརྒྱ་སྐྱེད་ལྟེ་བ | | | | | | |
|  |  | | སྒོ་ནོར་སྨན་ཁང་ | | | | | | |
|  |  | | ང་རང་གིས་སྒོ་ནོར་གསོ་བའི་འཕྲོད་སྟོན་ཚུ་བྱིནམ་ཨིན་ | | | | | | |
|  |  | | གཞན་ | | | | | | |
| P60 | ཁྱོད་ཀྱི་གོང་གི་དྲི་བ་འདི་ནང་ལུ ་གཞན་ཟེར་གདམ་འཐུ་འབད་ཡོད་པ་ཅིན་ རྒྱབ་ཁུངས་བཀལ་གནང་ | |  | | | | | | |
| P61 | ཁྱོད་ཀྱི་ནོར་དང་གཡག་ཚུ་གིས་དོན་ལུ་ སྒོ་ནོར་གསོ་བའི་འཕྲོད་སྟོན་དང་གཅིག་ཁར་འབྲེལ་བ་ག་དེ་འབད་འཐབ་དོ་ | | བདུན་ཕྲག་བཞིན་དུ | | | | | | |
|  |  | | ཟླ་རིམ་བཞིན་དུ | | | | | | |
|  |  | | ལོ་ལྟར་བཞིན་དུ | | | | | | |
|  |  | | སྒོ་ནོར་ལུ་ན་ཚ་ཕོག་པའི་སྐབས་རྐྱངམ་ཅིག | | | | | | |
|  |  | | ཞབས་ཏོག་ཞུ་ནི་རང་མེད་ | | | | | | |
| P62 | ཁྱོད་ལུ་ཞུ་བ་བཀོད་ཞིནམ་ལས་ སྒོ་ནོར་གསོ་བའི་ཞབས་ཏོག་ཕུལ་མི་ཚུ་གིས་ ཁྱོད་ཀྱི་ནོར་དང་གཡག་ཚུ་ཞིབ་དཔྱད་འབད་བཅུག་འོང་ག? | | འབད་བཅུག་འོང་ | | | | | | |
|  |  | | འབད་མི་བཅུག | | | | | | |
| P63 | ཁྱོད་ཀྱི་ནོར་དང་གཡག་ཚུ་གིས་ བརྟག་ཚན་ གཟུགས་ཀྱི་ལྗིད་ཚད་མར་ཕབས་ དྲོད་འབར་ སྤུ་བཙུབ་དྲགས་འབད་མཐོང་མ་ཅིན་ ག་ཅིག་འབད་འོང་? | | སྒོ་ནོར་གྱི་ཞབས་ཏོག་ཕུལ་མི་ཚུ་དང་གཅིག་ཁར་གྲོས་བསྟུན་འབད་དགོ | | | | | | |
|  |  | | སེམས་ཅན་འདི་ཁྲོམ་ཁར་བརྩོང་དགོ་ | | | | | | |
|  |  | | ང་རང་གི་སྨན་བཅོས་འབད་ནི་ | | | | | | |
|  |  | | ན་ཚ་གིས་བརྟགས་ཚན་བཏོན་ཡོད་པའི་སེམས་ཅན་འདི་ཟུར་གནས་ནང་བཞག་དགོ་ | | | | | | |
|  |  | | ག་ཅིག་ཡང་མི་འབད | | | | | | |
| P64 | ཁྱོད་ཀྱི་ཐད་དཀར་དུ་ནོར་དང་གཡག་དང་གཅིག་ཁར་འབྲེལ་བ་ག་དེ་འབད་ཡོད? (བོའུ་ཅུང་ཚརཝ་དང་ ཨོམ་ཞོ་མི་ སྨན་བཅོས་འབད་བའི་སྐབས་ སེམས་ཅན་གཙང་དྲགས་བཏོན་འདི་འཁྱུ་བའི་སྐབས) | | ཉིན་ལྟར་ | | | | | | |
|  |  | | བདུན་ཕྲག་ཅིག་ནང་ཚར་ཅིག་ | | | | | | |
|  |  | | བདུན་ཕྲག་ཅིག་ནང་བརྒྱ་ལམ་ཅིག་ | | | | | | |
|  |  | | ཟླ་རིམ་ | | | | | | |
|  |  | | ལོ་ལྟར་ | | | | | | |
|  |  | | ཐད་དཀར་དུ་འབྲེལ་བ་རང་མེད་ | | | | | | |

**Appendix A – survey questionnaire in English**

| **KAP Farmer** | *Page 1* |
| --- | --- |
|  |
| Please complete the survey below. |  |
| Thank you! |  |
|  |  |
| Survey Elapsed Time |  |
|  |  |
| What is your name? | __________________________________ |
|  |
|  |  |
| What is your sex? | Male |
|  | Female |
|  | Prefer not to say |
|  |  |
| Age | __________________________________ |
| Prefer not to say: please state prefer not to say |
|  |
|  |  |
| Which GEOG do you live in? | __________________________________ |
| Prefer not to say: please type prefer not to say |
|  |
|  |  |
| Educational level | Primary (1-6) |
|  | Middle (7-8) |
|  | Lower secondary (9-10) |
|  | Upper secondary (11-12) |
|  | Tertiary |
|  |  |
| How many people live in your household? (ie. regularly | __________________________________ |
| sleep in the same house) |
|  |  |
| How many years have you spent commercially farming? | Between 0 and 5 years |
|  | Between 5-10 years |
|  | Between 10-15 years |
|  | Between 15-20 years |
|  | More than 20 years |


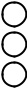

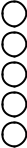

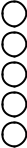


| Do you own any livestock? | Cattle |
| --- | --- |
|  | Goats |
|  | Pigs |

Yaks
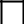
 Buffaloes

Sheep
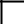

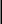
 Chickens

Donkeys

| If you own COWS/YAKS please indicate the total number | __________________________________ |
| --- | --- |
| you own. If you do not, enter 0. |

**This part of the KNOWLEDGE questionnaire is about tuberculosis in PEOPLE specifically**

| K1 | Have you heard/do you know about tuberculosis? | Yes |  |  |
| --- | --- | --- | --- | --- |
|  |  | No |  |  |
|  |  | I don't know | |  |
|  |  |  |  |  |
| K2 | Before taking this survey, were you aware that PEOPLE | Yes | No | I don't know |
|  | could be infected with tuberculosis? |  |  |  |


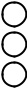

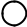

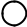

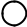


|  |  | *Page 2* |
| --- | --- | --- |
|  |  |  |
| K3 For the following question, check all that apply: What | | Direct contact with an infected person |
|  | are some ways that HUMANS can become infected with | Direct contact with an infected cow/yak/animal |
|  | tuberculosis? | Consumption of raw milk |
|  |  | Consumption of yoghurt made from raw milk |
|  |  | Consumption of cheese made from raw milk |
|  |  | Consumption of undercooked meat |
|  |  | Through contaminated wounds |
|  |  | Handling of raw birth material (eg. Placenta) |
|  |  | I don't know |
|  |  |  |
| K4 For the following question, check all that apply: What | | Enlarged/swollen neck area (lymph nodes) |
|  | are the signs and symptoms of a PERSON infected with | Cough |
|  | tuberculosis? | Cough lasting more than 3 weeks |
|  |  | Chest pain |
|  |  | Fever |
|  |  | Loss of appetite |
|  |  | Weight loss |
|  |  | I don't know |

**Based on your knowledge, to what extent do you agree with the following:**

|  | Strongly disagree | Disagree | Agree | Strongly agree | |  |
| --- | --- | --- | --- | --- | --- | --- |
| K5 | PEOPLE infected with |  |  |  |  |  |
|  | tuberculosis may look healthy |  |  |  |  |  |
| K6 | PEOPLE infected with |  |  |  |  |  |
|  | tuberculosis can be treated and |  |  |  |  |  |
|  | possibly cured |  |  |  |  |  |
| K7 | Testing for tuberculosis in |  |  |  |  |  |
|  | PEOPLE is available in Bhutan |  |  |  |  |  |
| K8 | Treatment for PEOPLE with |  |  |  |  |  |
|  | tuberculosis is available in |  |  |  |  |  |
| K9 | Bhutan |  |  |  |  |  |
|  | Vaccination against tuberculosis |  |  |  |  |  |
|  | is available for me in Bhutan |  |  |  |  |  |
|  |  |  | |  |  |  |
| K10 | What type of treatment is best to cure tuberculosis in | Modern/western medicine | |  |  |  |
|  | PEOPLE? | Traditional/Ayurvedic medicine | |  |  |  |
|  |  | Both |  |  |  |  |
|  |  | Cannot be cured | |  |  |  |
|  |  | I don't know |  |  |  |  |
|  |  |  | | |  | |
| K11 | For the following question, check all that apply: What | Reduce contact with a tuberculosis infected person | | | | |
|  | actions can you take to prevent becoming infected with | Frequently wash your hands | |  |  |  |
|  | tuberculosis? | Consume yoghurt made from boiled milk | | | | |
|  |  | Avoid close contact with sick cattle (eg. | | | | |
|  |  | touching, milking etc) | |  |  |  |
|  |  | Consume well-cooked meat | |  |  |  |
|  |  | Nothing |  |  |  |  |
|  |  |  | | |  | |
| K12 | For the following question, check all that apply: What | Covering mouth and nose when coughing | | | | |
|  | methods can be used to reduce the transmission of | Vaccination |  |  |  |  |
|  | tuberculosis between PEOPLE? | Isolating patients | |  |  |  |
|  |  | Avoid sharing of utensils | |  |  |  |


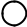

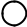

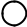

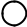

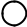

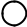

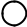

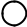

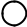

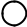

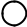

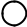

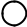

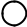

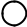

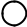

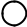

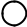

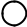

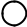

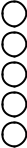


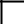

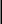
 Early treatment of patients


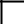

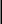
 Eating a balanced diet

*Page 3*

**This part of the KNOWLEDGE questionnaire is about tuberculosis in COWS/YAKS specifically bovine tuberculosis**

| K13 Before taking this survey did you know that people can | | Yes |
| --- | --- | --- |
|  | get tuberculosis from COWS/YAKS? (bovine tuberculosis) | No |
|  |  | I don't know |
|  |  |  |
| K14 | For the following question, check all that apply: How | Transmission of microorganisms/bacteria through |
|  | do you understand COWS/YAKS become infected with | the air |
|  | tuberculosis? | Digestion/absorption of microorganisms/bacteria |
|  |  | Direct contact with another infected animal |
|  |  | None of the above |
|  |  | I don't know |
|  |  |  |
| K15 | For the following question, check all that apply: What | Fever |
|  | are the signs a COW/YAK displays when infected with | Loss of body weight |
|  | tuberculosis? | Rough hair coat |
|  |  | Persistent coughing (a few weeks) |
|  |  | Shortness of breath |
|  |  | Nasal discharge |
|  |  | Enlarged/swollen neck area (lymph nodes) |
|  |  | There may be no signs |
|  |  | None of the above |
|  |  | I don't know |


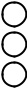


**Based on your knowledge, to what extent do you agree with the following:**

|  | Strongly disagree | Disagree | | Agree | Strongly agree | |
| --- | --- | --- | --- | --- | --- | --- |
| K16 | COWS/YAKS infected with bovien |  |  |  |  |  |
|  | tuberculosis may look healthy |  |  |  |  |  |
| K17 | COWS/YAKS infected with bovine |  |  |  |  |  |
|  | tuberculosis may survive for |  |  |  |  |  |
|  | many years |  |  |  |  |  |
| K18 | COWS/YAKS infected with bovine |  |  |  |  |  |
|  | tuberculosis may fully recover by |  |  |  |  |  |
|  | themselves without treatment |  |  |  |  |  |
| K19 | COWS/YAKS infected may die of |  |  |  |  |  |
|  | bovine tuberculosis |  |  |  |  |  |
|  |  |  |  |  |  |  |
| K20 | For the following question, check all that apply: What |  | Vaccination |  |  |  |
|  | can be done to reduce the transmission of bovine |  | Conduct tuberculin skin tests (TST) | | | |
|  | tuberculosis among COWS/YAKS? |  | Isolate infected animals | |  |  |
|  |  |  | Nothing |  |  |  |
|  |  |  | I don't know |  |  |  |
|  |  | |  |  |  |  |
| K21 | Do you know COW/YAK movement/purchase of a new COW/YAK | | Yes |  |  |  |
|  | is responsible for bovine tuberculosis transmission on |  | No |  |  |  |
|  | your farm? |  | I don't know |  |  |  |


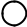

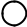

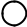

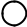

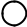

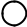

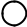

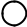

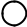

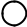

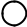

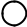

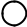

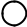

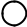

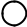

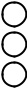


*Page 4*

**This part of the ATTITUDE questionnaire is about tuberculosis in PEOPLE specifically**

**Based on your attitude/perception, to what extent do you agree with the following:**

Strongly disagree Disagree Agree Strongly agree

A22 Vaccinating PEOPLE in Bhutan


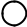

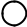

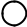

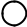


will protect me and my

community against tuberculosis

A23 A person with signs of


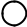

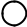

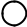

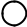


tuberculosis should seek medical

attention immediately

A24 Tuberculosis is an important


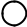

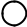

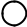

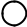


public health problem in Bhutan

A25 Tuberculosis only affects people


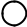

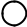

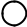

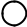


who are poor

| A26 | What is your predominant feeling towards PEOPLE | A lot of compassion |
| --- | --- | --- |
|  | infected with tuberculosis? | Somewhat compassionate |
|  |  | I am neutral, I have no particular feeling |
|  |  | Fearful of them |
|  |  |  |
| A27 | A PERSON with tuberculosis may experience... | Financial hardship |
|  |  | Health hardship |
|  |  | Work hardship |
|  |  | Social hardship |
|  |  | None of the above |
|  |  | Other |
|  |  |  |
| A28 | If you said 'other' to the previous question, please |  |
|  | explain what was meant | __________________________________________ |
|  |  |


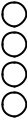

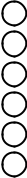


**This part of the ATTITUDE questionnaire is about tuberculosis in COWS/YAKS specifically bovine tuberculosis**

| A29 For the following question, check all that apply: | Dairy farmers |
| --- | --- |
| Which group/groups of people do you think are the most | Dairy traders |
| likely to become infected with bovine tuberculosis? | Dairy consumers |
|  | All of them |
|  | None |
|  | I don't know |

**Based on your attitude/perception, to what extent do you agree with the following:**

Strongly disagree Disagree Agree Strongly agree

A30 Having contact with COWS/YAKS


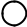

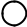

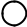

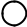


increases the risk of getting

infected with bovine tuberculosis

A31

|  |  | *Page 5* | |
| --- | --- | --- | --- |
|  | Consuming raw milk products |  |  |
|  | (milk, butter, cheese, yogurt etc) |  |  |
|  | increases the risk of being |  |  |
|  | infected with bovine tuberculosis |  |  |
| A32 | The consumption of raw animal |  |  |
|  | product (eg. beef) exposes |  |  |
|  | people to bovine tuberculosis |  |  |
| A33 | COWS/YAKS that appear sick |  |  |
|  | should be isolated from the rest |  |  |
|  | of the herd |  |  |
| A34 | My COWS/YAKS are at risk of |  |  |
|  | becoming sick with bovine |  |  |
|  | tuberculosis |  |  |
|  |  |  |  |
| A35 | For the following statement, check all that apply: If | be consumed by people | |
|  | a COW/YAK has died from any disease the COW/YAK | not be consumed and be buried in land and covered | |
|  | should... (does not appear to die of natural causes | not be consumed and be left to decompose naturally | |
|  | such as old age or an apparent injury) | I don't know | |
|  |  |  |  |
| A36 | For the following question, check all that apply: What | Sell the animal | |
|  | would you do if one of your COWS/YAKS are confirmed as | Treat the animal yourself | |
|  | tuberculosis positive? | Seek veterinary assistance | |
|  |  | Other | |
|  |  |  |  |
| A37 | If you said 'other' to the previous question, please |  |  |
|  | explain what was meant | __________________________________________ |  |
|  |  |  |


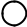

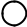

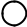

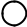

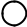

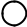

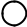

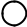

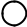

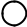

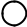

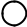

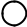

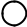

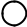

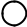


**This part of the PRACTICE questionnaire is about tuberculosis in PEOPLE specifically**

| P38 | What is YOUR most common habit of drinking milk? | Raw milk (including fresh milk and yogurt) |
| --- | --- | --- |
|  |  | Boiled |
|  |  | Pasteurised |
|  |  | Milk powder |
|  |  | I don't consume milk |
|  |  |  |
| P39 | If you selected 'Boiled' to the previous question, why | To remove diseases |
|  | do YOU boil milk? | Because it tastes better |
|  |  | Because my family always has |
|  |  | I prefer it hot |


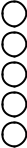

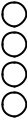


P40 How often do YOU drink milk?


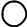
 Once a week


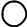
 Many times a week


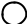
 Everyday


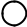
 Rarely (once on an odd occasion)

P41 How often do YOU consume dairy products (eg. cheese,
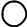
 Once a week

butter, yogurt)?
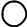
 Many times a week


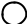
 Everyday

Rarely

Never

*Page 6*

| P42 | For the following question, check all that apply: | Homemade |
| --- | --- | --- |
|  | Where do YOU source your dairy products from? (eg. | Market bought |
|  | cheese, butter, yogurt) | Family/friend |
|  |  | Locally |
|  |  | Other |
|  |  |  |
| P43 | If you answered 'other' to the previous question, |  |
|  | please expand | __________________________________________ |
|  |  |
|  |  |  |
| P44 | What is YOUR most common habit of beef consumption? | Raw beef |
|  |  | Cooked beef |
|  |  | I don't consume beef |
|  |  |  |
| P45 | Where do YOU source your beef from? | Market |
|  |  | Family/friend |
|  |  | Locally (eg. neighbour) |
|  |  | Other |
|  |  |  |
| P46 | If you answered 'other' to the previous question, |  |
|  | please expand | __________________________________________ |
|  |  |
|  |  |  |
| P47 | Do YOU drink water from the same source as the water | Yes |
|  | you provide your COWS/YAKS? | No |
|  | eg. Do you collect water to drink from the same water | I don't know |
|  |  |
|  | source your COW/YAK drink from? |  |
|  |  |  |
| P48 | What would you do if you suspect YOU are infected with | Go to the hospital |
|  | tuberculosis? | Go to local health centre |
|  |  | Go to a traditional healer |
|  |  | Do nothing |
|  |  | Other |
|  |  |  |
| P49 | If you said 'other' to the previous question, please |  |
|  | explain what was meant | __________________________________________ |
|  |  |
|  |  |  |
| P50 | Do YOU wash your hands after having contact with/or | Never |
|  | milking COWS/YAKS? | Rarely |
|  |  | Sometimes |
|  |  | Always |
|  |  | I don't know |
|  |  |  |
| P51 | Have YOU ever tested yourself for tuberculosis? | Yes |
|  |  | No |
|  |  | I don't know |
|  |  |  |
| P52 | Have YOU ever received treatment for tuberculosis? | Yes |
|  |  | No |
|  |  | I don't know |
|  |  |  |
| P53 | What is YOUR families vaccination status against | I am vaccinated |
|  | tuberculosis (bacille Calmette-Guérin vaccine)? | My child/children are vaccinated |
|  |  | My entire family is vaccinated |
|  |  | I don't know |

*Page 7*

P54

Do YOU get regular medical check-ups (at least once a year)?

Yes

No

**This part of the PRACTICE questionnaire is about FARMING PRACTICES**

**Please answer YES or NO to the following:**

|  | Yes | No | |
| --- | --- | --- | --- |
| P55 | Do YOU use any protective |  |  |
|  | equipment while working on the |  |  |
|  | farm? (masks, gloves, gumboot, |  |  |
|  | apron) |  |  |
| P56 | Do YOU practice daily cleaning |  |  |
|  | and sanitation practices in your |  |  |
|  | farm? (regular cleaning cow |  |  |
|  | dung in stable, washing floor |  |  |
|  | with detergents) |  |  |
| P57 | Do YOU wash your hands |  |  |
|  | immediately before and after |  |  |
|  | working in the dairy farm? |  |  |
| P58 | Do YOU always protect your |  |  |
|  | wounds while handling or |  |  |
|  | working with COWS/YAKS? |  |  |
|  |  |  |  |
| P59 | Who provides veterinary health care for your | Livestock extension centre | |
|  | COWS/YAKS? | Veterinary hospital | |
|  |  | I provide healthcare to my animals | |
|  |  | Other | |
|  |  |  |  |
| P60 | If you answered 'other' to the previous question, |  |  |
|  | please expand | __________________________________________ |  |
|  |  |  |
|  |  |  |  |
| P61 | How often do you seek healthcare for your COWS/YAKS? | Weekly | |
|  |  | Monthly | |
|  |  | Annually | |
|  |  | When they appear sick | |
|  |  | Never | |
|  |  |  |  |
| P62 | On request, will you allow inspection of your | Yes | |
|  | COWS/YAKS by a veterinary service? | No | |
|  |  |  |  |
| P63 | What do you do when your COWS/YAKS are displaying | Consult veterinary service | |
|  | symptoms such as weight loss, fever and/or rough hair | Sell animal in market | |
|  | coat | I treat them myself | |
|  |  | Isolate infected animal/s | |
|  |  | Do nothing | |

P64

How often do YOU have direct contact with COWS/YAKS? (calving, milking, providing healthcare, cleaning animals)

Daily

Once a week

A few times a week

Monthly

Yearly

Never

*Page 8*

**Appendix B: Frequencies of knowledge, attitudes, and practices stratified by Dzongkhags.**

*Table 1: Bhutanese dairy farmers’ responses to knowledge-based questions by dzongkhag.*

| **Question** | **Responses** | **Haa**,  N = 133 | **Paro**,  N = 46 | **Thimphu**,  N = 85 | **Overall**,  N = 264 |
| --- | --- | --- | --- | --- | --- |
| How can humans become infected with bovine tuberculosis? (select all that apply) | Direct contact with an infected person | 17 (13.0%) | 0 (0.0%) | 50 (58.8%) | 67 (25.6%) |
|  | Direct contact with infected cattle | 38 (29.0%) | 5 (10.9%) | 58 (68.2%) | 101 (38.5%) |
|  | Consumption of raw milk | 15 (11.5%) | 3 (6.5%) | 57 (67.1%) | 75 (28.6%) |
|  | Consumption of unpasteurised or undercooked animal products | 3 (2.3%) | 6 (13.0%) | 66 (77.6%) | 75 (28.6%) |
|  | I don’t know | 89 (67.9%) | 37 (80.4%) | 6 (7.1%) | 132 (50.4%) |
|  | Missing* | 2 | 0 | 0 | 2 |
| What are the signs and symptoms that a PERSON infected with bovine tuberculosis could display? (select all that apply) | Enlarged/swollen neck area (lymph nodes) | 1 (0.8%) | 2 (4.3%) | 9 (10.7%) | 12 (4.6%) |
|  | Persistent cough | 43 (32.3%) | 4 (8.7%) | 59 (70.2%) | 106 (40.3%) |
|  | Chest pain | 5 (3.8%) | 5 (10.9%) | 51 (60.7%) | 61 (23.2%) |
|  | Fever | 3 (2.3%) | 8 (17.4%) | 43 (51.2%) | 54 (20.5%) |
|  | Loss of appetite | 47 (35.3%) | 8 (17.4%) | 54 (64.3%) | 109 (41.4%) |
|  | Weight loss | 44 (33.1%) | 4 (8.7%) | 57 (67.9%) | 105 (39.9%) |
|  | I don’t know | 83 (62.4%) | 38 (82.6%) | 6 (7.1%) | 127 (48.3%) |
|  | Missing* | 0 | 0 | 1 | 1 |
| PEOPLE infected with any type of tuberculosis may look healthy | Yes | 24 (18.0%) | 5 (10.9%) | 1 (1.2%) | 30 (11.4%) |
|  | No | 99 (74.4%) | 30 (65.2%) | 83 (98.8%) | 212 (80.6%) |
|  | I don’t know | 10 (7.5%) | 11 (23.9%) | 0 (0.0%) | 21 (8.0%) |
|  | Missing* | 0 | 0 | 1 | 1 |
| PEOPLE infected with any type of tuberculosis can be cured | Yes | 89 (67.4%) | 33 (71.7%) | 54 (63.5%) | 176 (66.9%) |
|  | No | 28 (21.2%) | 2 (4.3%) | 28 (32.9%) | 58 (22.1%) |
|  | I don’t know | 15 (11.4%) | 11 (23.9%) | 3 (3.5%) | 29 (11.0%) |
|  | Missing* | 1 | 0 | 0 | 1 |
| PEOPLE can get treatment against tuberculosis in Bhutan | Yes | 114 (85.7%) | 33 (73.3%) | 84 (98.8%) | 231 (87.8%) |
|  | No | 14 (10.5%) | 0 (0.0%) | 1 (1.2%) | 15 (5.7%) |
|  | I don’t know | 5 (3.8%) | 12 (26.7%) | 0 (0.0%) | 17 (6.5%) |
|  | Missing* | 0 | 1 | 0 | 1 |
| PEOPLE can get a vaccine against tuberculosis in Bhutan | Yes | 34 (25.6%) | 29 (64.4%) | 61 (71.8%) | 124 (47.1%) |
|  | No | 18 (13.5%) | 0 (0.0%) | 3 (3.5%) | 21 (8.0%) |
|  | I don’t know | 81 (60.9%) | 16 (35.6%) | 21 (24.7%) | 118 (44.9%) |
|  | Missing* | 0 | 1 | 0 | 1 |
| Vaccination can protect people against bovine tuberculosis | Yes | 72 (54.1%) | 31 (67.4%) | 40 (47.1%) | 143 (54.2%) |
|  | No | 11 (8.3%) | 2 (4.3%) | 28 (32.9%) | 41 (15.5%) |
|  | I don’t know | 50 (37.6%) | 13 (28.3%) | 17 (20.0%) | 80 (30.3%) |
| Human tuberculosis is an important public health problem in Bhutan | Yes | 64 (48.5%) | 37 (80.4%) | 75 (88.2%) | 176 (66.9%) |
|  | No | 20 (15.2%) | 1 (2.2%) | 10 (11.8%) | 31 (11.8%) |
|  | I don’t know | 48 (36.4%) | 8 (17.4%) | 0 (0.0%) | 56 (21.3%) |
|  | Missing* | 1 | 0 | 0 | 1 |
| How can cattle become infected with bovine tuberculosis (check only those you know) | Direct contact with an infected animal | 91 (68.4%) | 31 (67.4%) | 22 (25.9%) | 144 (54.5%) |
|  | Respiratory transmission (air droplets) | 16 (12.0%) | 6 (13.0%) | 57 (67.1%) | 79 (29.9%) |
|  | Oral transmission (contaminated feed) | 3 (2.3%) | 8 (17.4%) | 60 (70.6%) | 71 (26.9%) |
|  | I don’t know | 89 (66.9%) | 29 (63.0%) | 5 (5.9%) | 123 (46.6%) |
| What are the signs and symptoms that cattle infected with bovine tuberculosis could display? Check only those you know | Fever | 6 (4.5%) | 3 (6.5%) | 45 (54.2%) | 54 (20.6%) |
|  | Loss of body weight | 41 (30.8%) | 9 (19.6%) | 63 (75.9%) | 113 (43.1%) |
|  | Rough hair coat | 19 (14.3%) | 15 (32.6%) | 29 (34.9%) | 63 (24.0%) |
|  | Persistent cough (a few weeks) | 38 (28.6%) | 2 (4.3%) | 49 (59.0%) | 89 (34.0%) |
|  | Shortness of breath | 42 (31.6%) | 6 (13.0%) | 43 (51.8%) | 91 (34.7%) |
|  | Nasal discharge | 12 (9.0%) | 4 (8.7%) | 36 (43.4%) | 52 (19.8%) |
|  | I don’t know | 76 (57.1%) | 27 (58.7%) | 10 (12.0%) | 113 (43.1%) |
|  | Missing* | 0 | 0 | 2 | 2 |
| Cattle infected with bovine tuberculosis may look healthy | Yes | 19 (15.0%) | 0 (0.0%) | 2 (2.4%) | 21 (8.2%) |
|  | No | 94 (74.0%) | 23 (52.3%) | 77 (90.6%) | 194 (75.8%) |
|  | I don’t know | 14 (11.0%) | 21 (47.7%) | 6 (7.1%) | 41 (16.0%) |
|  | Missing* | 6 | 2 | 0 | 8 |
| Cattle infected with bovine tuberculosis may live for many years | Yes | 29 (21.8%) | 1 (2.2%) | 11 (12.9%) | 41 (15.5%) |
|  | No | 39 (29.3%) | 24 (52.2%) | 70 (82.4%) | 133 (50.4%) |
|  | I don’t know | 65 (48.9%) | 21 (45.7%) | 4 (4.7%) | 90 (34.1%) |
| Cattle infected with bovine tuberculosis may live for many years | Yes | 9 (6.9%) | 2 (4.3%) | 1 (1.2%) | 12 (4.6%) |
|  | No | 36 (27.5%) | 24 (52.2%) | 81 (95.3%) | 141 (53.8%) |
|  | I don’t know | 86 (65.6%) | 20 (43.5%) | 3 (3.5%) | 109 (41.6%) |
|  | Missing* | 2 | 0 | 0 | 2 |
| What can be done to reduce the transmission of bovine tuberculosis among cattle? Check only those you know | Vaccination | 79 (59.4%) | 18 (39.1%) | 18 (21.2%) | 115 (43.6%) |
|  | Tuberculin skin test | 0 (0.0%) | 0 (0.0%) | 0 (0.0%) | 0 (0.0%) |
|  | Isolate infected animals | 33 (24.8%) | 12 (26.1%) | 64 (75.3%) | 109 (41.3%) |
|  | Culling | 1 (0.8%) | 0 (0.0%) | 1 (1.2%) | 2 (0.8%) |
|  | I don’t know | 20 (15.0%) | 16 (34.8%) | 2 (2.4%) | 38 (14.4%) |

* Missing responses were absent from the dataset and were excluded from percentage calculations within each dzongkhag.

*Table 2: Bhutanese dairy farmers’ attitudes towards the risk of bTB by dzongkhag.*

| **Question** | **Response** | **Haa**,  N = 133 | **Paro**,  N = 46 | **Thimphu**,  N = 85 | **Overall**,  N = 264 |
| --- | --- | --- | --- | --- | --- |
| In Bhutan, what do you think is the risk of people getting infected with bovine tuberculosis from consuming milk? | Low risk | 46 (34.8%) | 14 (30.4%) | 26 (31.0%) | 86 (32.8%) |
|  | Medium risk | 49 (37.1%) | 18 (39.1%) | 26 (31.0%) | 93 (35.5%) |
|  | High risk | 37 (28.0%) | 14 (30.4%) | 32 (38.1%) | 83 (31.7%) |
|  | Missing* | 1 | 0 | 1 | 2 |
| In Bhutan, what do you think is the risk of people getting infected with bovine tuberculosis from consuming dairy products (e.g. cheese, butter, yogurt)? | Low risk | 31 (23.3%) | 14 (30.4%) | 26 (31.0%) | 71 (27.0%) |
|  | Medium risk | 74 (55.6%) | 22 (47.8%) | 28 (33.3%) | 124 (47.1%) |
|  | High risk | 28 (21.1%) | 10 (21.7%) | 30 (35.7%) | 68 (25.9%) |
|  | Missing* | 0 | 0 | 1 | 1 |
| In Bhutan, what do you think is the risk of people getting infected with bovine tuberculosis from consuming meat? | Low risk | 12 (9.0%) | 10 (21.7%) | 15 (18.1%) | 37 (14.1%) |
|  | Medium risk | 40 (30.1%) | 16 (34.8%) | 23 (27.7%) | 79 (30.2%) |
|  | High risk | 81 (60.9%) | 20 (43.5%) | 45 (54.2%) | 146 (55.7%) |
|  | Missing* | 0 | 0 | 2 | 2 |
| In Bhutan, what do you think is the risk of people getting infected with bovine tuberculosis from consuming an animal with signs of bovine tuberculosis? | Low risk | 10 (7.5%) | 1 (2.2%) | 4 (4.8%) | 15 (5.7%) |
|  | Medium risk | 34 (25.6%) | 2 (4.3%) | 5 (6.0%) | 41 (15.6%) |
|  | High risk | 89 (66.9%) | 43 (93.5%) | 75 (89.3%) | 207 (78.7%) |
|  | Missing* | 0 | 0 | 1 | 1 |
| In Bhutan, what do you think is the risk of people getting infected with bovine tuberculosis from contact with animals? | Low risk | 20 (15.0%) | 9 (19.6%) | 16 (19.0%) | 45 (17.1%) |
|  | Medium risk | 76 (57.1%) | 27 (58.7%) | 18 (21.4%) | 121 (46.0%) |
|  | High risk | 37 (27.8%) | 10 (21.7%) | 50 (59.5%) | 97 (36.9%) |
|  | Missing* | 0 | 0 | 1 | 1 |
| In Bhutan, what do you think is the risk of people getting infected with bovine tuberculosis from contact with people? | Low risk | 31 (23.3%) | 18 (39.1%) | 13 (15.5%) | 62 (23.6%) |
|  | Medium risk | 54 (40.6%) | 25 (54.3%) | 25 (29.8%) | 104 (39.5%) |
|  | High risk | 48 (36.1%) | 3 (6.5%) | 46 (54.8%) | 97 (36.9%) |
|  | Missing* | 0 | 0 | 1 | 1 |
| In Bhutan, what do you think is the risk of people getting infected with bovine tuberculosis from allowing cattle into your dwelling/living area | Low risk | 9 (6.8%) | 10 (21.7%) | 9 (10.8%) | 28 (10.7%) |
|  | Medium risk | 41 (30.8%) | 27 (58.7%) | 25 (30.1%) | 93 (35.5%) |
|  | High risk | 83 (62.4%) | 9 (19.6%) | 49 (59.0%) | 141 (53.8%) |
|  | Missing* | 0 | 0 | 2 | 2 |
| In Bhutan, what do you think is the risk of people getting infected with bovine tuberculosis from sharing a water source with your cattle | Low risk | 21 (15.9%) | 8 (17.4%) | 11 (13.3%) | 40 (15.3%) |
|  | Medium risk | 71 (53.8%) | 26 (56.5%) | 16 (19.3%) | 113 (43.3%) |
|  | High risk | 40 (30.3%) | 12 (26.1%) | 56 (67.5%) | 108 (41.4%) |
|  | Missing* | 1 | 0 | 2 | 3 |
| What do you think is the risk of infection from bovine tuberculosis among dairy farmers? | Low risk | 25 (18.8%) | 8 (17.4%) | 15 (17.6%) | 48 (18.2%) |
|  | Medium risk | 29 (21.8%) | 17 (37.0%) | 9 (10.6%) | 55 (20.8%) |
|  | High risk | 79 (59.4%) | 21 (45.7%) | 61 (71.8%) | 161 (61.0%) |
| What do you think is the risk of infection from bovine tuberculosis among cattle traders? | Low risk | 10 (7.5%) | 1 (2.2%) | 10 (11.8%) | 21 (8.0%) |
|  | Medium risk | 80 (60.2%) | 11 (23.9%) | 16 (18.8%) | 107 (40.5%) |
|  | High risk | 43 (32.3%) | 34 (73.9%) | 59 (69.4%) | 136 (51.5%) |
| What do you think is the risk of infection from bovine tuberculosis among dairy consumers? | Low risk | 10 (7.5%) | 9 (19.6%) | 14 (16.5%) | 33 (12.5%) |
|  | Medium risk | 62 (46.6%) | 19 (41.3%) | 36 (42.4%) | 117 (44.3%) |
|  | High risk | 61 (45.9%) | 18 (39.1%) | 35 (41.2%) | 114 (43.2%) |
| What do you think is the risk of infection from bovine tuberculosis among meat consumers? | Low risk | 3 (2.3%) | 9 (19.6%) | 6 (7.1%) | 18 (6.8%) |
|  | Medium risk | 23 (17.3%) | 15 (32.6%) | 30 (35.3%) | 68 (25.8%) |
|  | High risk | 107 (80.5%) | 22 (47.8%) | 49 (57.6%) | 178 (67.4%) |
| What do you think is the risk of infection from bovine tuberculosis among Veterinarians/Paraveterinarians? | Low risk | 56 (42.1%) | 32 (69.6%) | 37 (43.5%) | 125 (47.3%) |
|  | Medium risk | 35 (26.3%) | 5 (10.9%) | 28 (32.9%) | 68 (25.8%) |
|  | High risk | 42 (31.6%) | 9 (19.6%) | 20 (23.5%) | 71 (26.9%) |
| What would you do if one of your cattle has a rough coat, persistent cough and a loss of body weight? Choose one option | Sell the animal | 0 (0.0%) | 0 (0.0%) | 0 (0.0%) | 0 (0.0%) |
|  | Isolate the animal | 1 (0.8%) | 14 (30.4%) | 14 (16.7%) | 29 (11.0%) |
|  | Seek veterinary assistance | 132 (99.2%) | 32 (69.6%) | 70 (83.3%) | 234 (89.0%) |
|  | Cull the animal | 0 (0.0%) | 0 (0.0%) | 0 (0.0%) | 0 (0.0%) |
|  | Nothing | 0 (0.0%) | 0 (0.0%) | 0 (0.0%) | 0 (0.0%) |
|  | Missing* | 0 | 0 | 1 | 1 |
| In Bhutan, what is the risk of bovine tuberculosis infection when buying new cattle into your herd? | Low risk | 36 (27.1%) | 3 (6.5%) | 22 (25.9%) | 61 (23.1%) |
|  | Medium risk | 53 (39.8%) | 29 (63.0%) | 26 (30.6%) | 108 (40.9%) |
|  | High risk | 44 (33.1%) | 14 (30.4%) | 37 (43.5%) | 95 (36.0%) |
| In Bhutan, what is think the risk of bovine tuberculosis infection when there is contact between your cattle and neighbouring herds? | Low risk | 21 (15.8%) | 2 (4.3%) | 12 (14.1%) | 35 (13.3%) |
|  | Medium risk | 83 (62.4%) | 26 (56.5%) | 34 (40.0%) | 143 (54.2%) |
|  | High risk | 29 (21.8%) | 18 (39.1%) | 39 (45.9%) | 86 (32.6%) |
| In Bhutan, what is think the risk of bovine tuberculosis infection when there is contact between your cattle and veterinarians/paraveterinarians? | Low risk | 41 (30.8%) | 19 (41.3%) | 25 (29.4%) | 85 (32.2%) |
|  | Medium risk | 66 (49.6%) | 21 (45.7%) | 45 (52.9%) | 132 (50.0%) |
|  | High risk | 26 (19.5%) | 6 (13.0%) | 15 (17.6%) | 47 (17.8%) |
| In Bhutan, what is think the risk of bovine tuberculosis infection when there is contact between your cattle and wildlife | Low risk | 32 (24.1%) | 17 (37.0%) | 19 (22.4%) | 68 (25.8%) |
|  | Medium risk | 66 (49.6%) | 16 (34.8%) | 42 (49.4%) | 124 (47.0%) |
|  | High risk | 35 (26.3%) | 13 (28.3%) | 24 (28.2%) | 72 (27.3%) |
| In Bhutan, what is think the risk of bovine tuberculosis infection when there is contact between your cattle and other livestock species | Low risk | 50 (37.9%) | 13 (28.3%) | 20 (23.5%) | 83 (31.6%) |
|  | Medium risk | 55 (41.7%) | 19 (41.3%) | 40 (47.1%) | 114 (43.3%) |
|  | High risk | 27 (20.5%) | 14 (30.4%) | 25 (29.4%) | 66 (25.1%) |
|  | Missing* | 1 | 0 | 0 | 1 |
| In Bhutan, what is think the risk of bovine tuberculosis infection when there is contact between your cattle and dogs | Low risk | 73 (54.9%) | 19 (42.2%) | 23 (27.1%) | 115 (43.7%) |
|  | Medium risk | 41 (30.8%) | 15 (33.3%) | 29 (34.1%) | 85 (32.3%) |
|  | High risk | 19 (14.3%) | 11 (24.4%) | 33 (38.8%) | 63 (24.0%) |
|  | Missing* | 0 | 1 | 0 | 1 |

* Missing responses were absent from the dataset and were excluded from percentage calculations within each dzongkhag.

*Table 3: Bhutanese dairy farmers’ bTB-related practices by dzongkhag.*

| **Questions** | **Response** | **Haa**,  N = 133 | **Paro**,  N = 46 | **Thimphu**,  N = 85 | **Overall**,  N = 264 |
| --- | --- | --- | --- | --- | --- |
| What type of milk do you consume most often? | Boiled (less than 10mins) | 72 (54.1%) | 6 (21.4%) | 16 (19.0%) | 94 (38.4%) |
|  | Boiled (more than 10mins) | 47 (35.3%) | 10 (35.7%) | 27 (32.1%) | 84 (34.3%) |
|  | I dont consume milk | 0 (0.0%) | 3 (10.7%) | 3 (3.6%) | 6 (2.4%) |
|  | Milk powder | 0 (0.0%) | 8 (28.6%) | 34 (40.5%) | 42 (17.1%) |
|  | Pasteurised milk in tetra pack | 1 (0.8%) | 1 (3.6%) | 3 (3.6%) | 5 (2.0%) |
|  | Raw/fresh | 13 (9.8%) | 0 (0.0%) | 1 (1.2%) | 14 (5.7%) |
|  | Missing* | 0 | 18 | 1 | 19 |
| How often do you consume milk? | Always | 128 (96.2%) | 3 (10.7%) | 32 (38.1%) | 163 (66.5%) |
|  | Frequently | 5 (3.8%) | 8 (28.6%) | 17 (20.2%) | 30 (12.2%) |
|  | Never | 0 (0.0%) | 3 (10.7%) | 3 (3.6%) | 6 (2.4%) |
|  | Rarely | 0 (0.0%) | 14 (50.0%) | 32 (38.1%) | 46 (18.8%) |
|  | Missing* | 0 | 18 | 1 | 19 |
| How often do you consume dairy products (cheese, yogurt, butter)? | Always | 126 (95.5%) | 26 (56.5%) | 58 (68.2%) | 210 (79.8%) |
|  | Frequently | 6 (4.5%) | 16 (34.8%) | 15 (17.6%) | 37 (14.1%) |
|  | Never | 0 (0.0%) | 1 (2.2%) | 0 (0.0%) | 1 (0.4%) |
|  | Rarely | 0 (0.0%) | 3 (6.5%) | 12 (14.1%) | 15 (5.7%) |
|  | Missing* | 1 | 0 | 0 | 1 |
| How often do you consume meat? | Always | 7 (5.3%) | 0 (0.0%) | 3 (3.7%) | 10 (3.9%) |
|  | Frequently | 113 (85.0%) | 21 (48.8%) | 5 (6.1%) | 139 (53.9%) |
|  | Never | 0 (0.0%) | 3 (7.0%) | 4 (4.9%) | 7 (2.7%) |
|  | Rarely | 13 (9.8%) | 19 (44.2%) | 70 (85.4%) | 102 (39.5%) |
|  | Missing* | 0 | 3 | 3 | 6 |
| Where do you source your milk from? | Don’t consume | 0 (0.0%) | 3 (10.7%) | 3 (3.6%) | 6 (2.4%) |
|  | Friend/family/neighbour | 0 (0.0%) | 2 (7.1%) | 0 (0.0%) | 2 (0.8%) |
|  | Homemade | 133 (100.0%) | 21 (75.0%) | 59 (70.2%) | 213 (86.9%) |
|  | Market | 0 (0.0%) | 2 (7.1%) | 22 (26.2%) | 24 (9.8%) |
|  | Missing* | 0 | 18 | 1 | 19 |
| Where do you source your dairy products (Cheese, butter, yogurt) from? | Don’t consume | 0 (0.0%) | 1 (2.2%) | 0 (0.0%) | 1 (0.4%) |
|  | Friend/family/neighbour | 0 (0.0%) | 6 (13.0%) | 0 (0.0%) | 6 (2.3%) |
|  | Homemade | 133 (100.0%) | 18 (39.1%) | 54 (63.5%) | 205 (77.7%) |
|  | Market | 0 (0.0%) | 21 (45.7%) | 31 (36.5%) | 52 (19.7%) |
| Where do you source your meat from? | Don’t consume | 0 (0.0%) | 3 (7.0%) | 4 (4.9%) | 7 (2.7%) |
|  | Friend/family/neighbour | 10 (7.5%) | 0 (0.0%) | 0 (0.0%) | 10 (3.9%) |
|  | Homemade | 8 (6.0%) | 0 (0.0%) | 0 (0.0%) | 8 (3.1%) |
|  | Market | 115 (86.5%) | 40 (93.0%) | 77 (95.1%) | 232 (90.3%) |
|  | Missing* | 0 | 3 | 4 | 7 |
| How often do you wash your hands after contact with cattle? | Always | 55 (41.4%) | 44 (95.7%) | 77 (90.6%) | 176 (66.7%) |
|  | Frequently | 75 (56.4%) | 1 (2.2%) | 5 (5.9%) | 81 (30.7%) |
|  | Rarely | 3 (2.3%) | 1 (2.2%) | 3 (3.5%) | 7 (2.7%) |
|  | Never | 0 (0.0%) | 0 (0.0%) | 0 (0.0%) | 0 (0.0%) |
| How often do you wash your hands before and after milking cattle? | Always | 111 (83.5%) | 46 (100.0%) | 80 (94.1%) | 237 (89.8%) |
|  | Frequently | 22 (16.5%) | 0 (0.0%) | 5 (5.9%) | 27 (10.2%) |
|  | Rarely | 0 (0.0%) | 0 (0.0%) | 0 (0.0%) | 0 (0.0%) |
|  | Never | 0 (0.0%) | 0 (0.0%) | 0 (0.0%) | 0 (0.0%) |
| How often do you wear protective equipment (e.g., gloves) when treating cattle? | Always | 16 (12.0%) | 8 (17.4%) | 31 (36.5%) | 55 (20.8%) |
|  | Frequently | 23 (17.3%) | 7 (15.2%) | 4 (4.7%) | 34 (12.9%) |
|  | Rarely | 54 (40.6%) | 26 (56.5%) | 20 (23.5%) | 100 (37.9%) |
|  | Never | 40 (30.1%) | 5 (10.9%) | 30 (35.3%) | 75 (28.4%) |
| How often do you wear protective equipment (e.g., gloves) when handling birth material (e.g., placenta)? | Always | 1 (0.8%) | 9 (19.6%) | 39 (45.9%) | 49 (18.6%) |
|  | Frequently | 7 (5.3%) | 4 (8.7%) | 5 (5.9%) | 16 (6.1%) |
|  | Rarely | 62 (46.6%) | 28 (60.9%) | 11 (12.9%) | 101 (38.3%) |
|  | Never | 63 (47.4%) | 5 (10.9%) | 30 (35.3%) | 98 (37.1%) |
| How often do you clean and sanitise milking tools before and after milking? | Always | 74 (55.6%) | 31 (67.4%) | 52 (61.2%) | 157 (59.5%) |
|  | Frequently | 51 (38.3%) | 4 (8.7%) | 22 (25.9%) | 77 (29.2%) |
|  | Rarely | 4 (3.0%) | 11 (23.9%) | 4 (4.7%) | 19 (7.2%) |
|  | Never | 4 (3.0%) | 0 (0.0%) | 7 (8.2%) | 11 (4.2%) |
| How often do you clean and sanitise the farm (e.g., wash floors with detergent)? | Always | 4 (3.0%) | 5 (10.9%) | 24 (28.6%) | 33 (12.5%) |
|  | Frequently | 24 (18.0%) | 8 (17.4%) | 10 (11.9%) | 42 (16.0%) |
|  | Rarely | 56 (42.1%) | 16 (34.8%) | 30 (35.7%) | 102 (38.8%) |
|  | Never | 49 (36.8%) | 17 (37.0%) | 20 (23.8%) | 86 (32.7%) |
|  | Missing* | 0 | 0 | 1 | 1 |
| Do you get at least one medical check-up per year? | Yes | 112 (88.2%) | 41 (89.1%) | 57 (67.1%) | 210 (81.4%) |
|  | No | 15 (11.8%) | 5 (10.9%) | 28 (32.9%) | 48 (18.6%) |
|  | Missing* | 6 | 0 | 0 | 6 |
| Have you ever been tested for tuberculosis? | Yes | 2 (1.5%) | 6 (13.0%) | 15 (17.6%) | 23 (8.7%) |
|  | No | 131 (98.5%) | 40 (87.0%) | 70 (82.4%) | 241 (91.3%) |
|  | I don’t know | 0 (0.0%) | 0 (0.0%) | 0 (0.0%) | 0 (0.0%) |
| Have you ever received treatment for tuberculosis? | Yes | 3 (2.3%) | 3 (6.5%) | 7 (8.2%) | 13 (4.9%) |
|  | No | 129 (97.0%) | 43 (93.5%) | 78 (91.8%) | 250 (94.7%) |
|  | I don’t know | 1 (0.8%) | 0 (0.0%) | 0 (0.0%) | 1 (0.4%) |
| Are you vaccinated against tuberculosis? | Yes | 1 (0.8%) | 6 (13.0%) | 14 (16.5%) | 21 (8.0%) |
|  | No | 132 (99.2%) | 23 (50.0%) | 37 (43.5%) | 192 (72.7%) |
|  | I don’t know | 0 (0.0%) | 17 (37.0%) | 34 (40.0%) | 51 (19.3%) |
| Who does primarily give healthcare to your cattle? Choose one option. | Veterinary service | 133 (100.0%) | 20 (43.5%) | 71 (83.5%) | 224 (84.8%) |
|  | Friend/family/neighbour | 0 (0.0%) | 20 (43.5%) | 7 (8.2%) | 27 (10.2%) |
|  | I do it myself | 0 (0.0%) | 6 (13.0%) | 7 (8.2%) | 13 (4.9%) |
|  | No healthcare | 0 (0.0%) | 0 (0.0%) | 0 (0.0%) | 0 (0.0%) |
| Reporting diseases in Bhutan is… | Very easy | 92 (69.2%) | 3 (6.5%) | 21 (24.7%) | 116 (43.9%) |
|  | Easy | 41 (30.8%) | 37 (80.4%) | 47 (55.3%) | 125 (47.3%) |
|  | Hard | 0 (0.0%) | 6 (13.0%) | 17 (20.0%) | 23 (8.7%) |
|  | Very hard | 0 (0.0%) | 0 (0.0%) | 0 (0.0%) | 0 (0.0%) |
| Getting assistance for a sick animal is… | Very easy | 89 (66.9%) | 5 (10.9%) | 20 (23.5%) | 114 (43.2%) |
|  | Easy | 44 (33.1%) | 30 (65.2%) | 38 (44.7%) | 112 (42.4%) |
|  | Hard | 0 (0.0%) | 11 (23.9%) | 27 (31.8%) | 38 (14.4%) |
|  | Very hard | 0 (0.0%) | 0 (0.0%) | 0 (0.0%) | 0 (0.0%) |

* Missing responses were absent from the dataset and were excluded from percentage calculations within each dzongkhag.

**Appendix C. Dimensional reduction and data clustering**

**Figure C1.** Scree Plot and Final Archetype dimensions. The proportion of variance explained by Archetypes 1 and 2 was 0.795 (standard deviation: 6.72) and 0.205 (standard deviation: 3.41), respectively

**Figure C2.** Elbow Plot and K-Means Clustering Visualisation. The elbow plot shows the within-cluster sum of squares across different cluster numbers, suggesting that two or three cluster solutions derived from K-means partitioning of the generalised low-rank model matrix could be optimal. The plots show that the two-cluster solution results in clusters characterised by the variables with higher loads on Archetype 1 and 2; the three-cluster solution results in two clusters (1 and 2) explained by variables with heavy loads on Archetype 2. As Archetype 1 explains most of the variability in the data, we accepted the two clusters solution.

**Fig C3.** Factors with high loads on archetype 1 (arch>=1 and arch2 < 0) and for archetype 2 (arch1 <0 and arch2 >=1. These factors characterise the archetypes which, in turn, explain different fractions of variability observed in the data.

**Table C1.** Characterisation of the k-means two-cluster solution. The variables used are the top loads on Archetype 1 (not presented in table 2 in the main manuscript)

| **Questions** | **Cluster 1**,  N = 134 | **Cluster 2**,  N = 130 |
| --- | --- | --- |
| Perceived risk of zoonotic bTB associated with sharing water source with cattle |  |  |
| Low risk | 18 (13.6%) | 22 (17.1%) |
| Medium risk | 28 (21.2%) | 85 (65.9%) |
| High risk | 86 (65.2%) | 22 (17.1%) |
| Missing* | 2 | 1 |
| Perceived risk of zoonotic bTB for cattle traders |  |  |
| Low risk | 10 (7.5%) | 11 (8.5%) |
| Medium risk | 28 (20.9%) | 79 (60.8%) |
| High risk | 96 (71.6%) | 40 (30.8%) |
| Can people get a vaccine against tuberculosis in Bhutan? |  |  |
| Yes | 87 (64.9%) | 37 (28.7%) |
| No | 5 (3.7%) | 16 (12.4%) |
| I dont know | 42 (31.3%) | 76 (58.9%) |
| Missing* | 0 | 1 |
| Cattle infected with bTB may fully recover without treatment |  |  |
| Yes | 6 (4.5%) | 6 (4.7%) |
| No | 101 (75.4%) | 40 (31.3%) |
| I dont know | 27 (20.1%) | 82 (64.1%) |
| Missing* | 0 | 2 |
| Cattle infected with bTB may live for many years |  |  |
| Yes | 15 (11.2%) | 26 (20.0%) |
| No | 92 (68.7%) | 41 (31.5%) |
| I dont know | 27 (20.1%) | 63 (48.5%) |
| Perceived risk of cow-to-cow bTB transmission due to contact with neighbouring cattle |  |  |
| Low risk | 13 (9.7%) | 22 (16.9%) |
| Medium risk | 48 (35.8%) | 95 (73.1%) |
| High risk | 73 (54.5%) | 13 (10.0%) |
| Perceived risk of zoonotic bTB for cattle farmers |  |  |
| Low risk | 17 (12.7%) | 31 (23.8%) |
| Medium risk | 12 (9.0%) | 43 (33.1%) |
| High risk | 105 (78.4%) | 56 (43.1%) |
| Perceived risk of zoonotic bTB associated with sharing allowing cattle into dwelling |  |  |
| Low risk | 17 (12.9%) | 11 (8.5%) |
| Medium risk | 31 (23.5%) | 62 (47.7%) |
| High risk | 84 (63.6%) | 57 (43.8%) |
| Missing* | 2 | 0 |
| Perceived risk of cow-to-cow bTB transmission due buying new cattle into the herd |  |  |
| Low risk | 24 (17.9%) | 37 (28.5%) |
| Medium risk | 38 (28.4%) | 70 (53.8%) |
| High risk | 72 (53.7%) | 23 (17.7%) |
